# Localized NF‐κB Inhibition Reduces Lipid Nanoparticle‐Associated Inflammation

**DOI:** 10.1002/advs.202517931

**Published:** 2026-03-24

**Authors:** Carolann L. Espy, Jichuan Wu, Serena Omo‐Lamai, Liuqian Wang, Fengyi Dong, Aleksa Milosavljevic, Sarah O'Neill, Taylor Brysgel, Yufei Wang, Michael Zaleski, Emily Wolfe, Rhea Maheshwari, Manthan Patel, Dirk Trauner, Jia Nong, Zhicheng Wang, Jacob S. Brenner

**Affiliations:** ^1^ Department of Systems Pharmacology and Translational Therapeutics Perelman School of Medicine University of Pennsylvania Philadelphia Pennsylvania USA; ^2^ Division of Pulmonary Allergy, and Critical Care Department of Medicine Perelman School of Medicine University of Pennsylvania Philadelphia Pennsylvania USA; ^3^ Department of Bioengineering University of Pennsylvania Philadelphia Pennsylvania USA; ^4^ School of Biomedical Engineering Science and Health Systems Drexel University Philadelphia Pennsylvania USA

**Keywords:** inflammation, nanoparticles, NF‐κB inhibition, small molecule drug loading

## Abstract

Lipid nanoparticles (LNPs) are widely used for nucleic acid delivery, yet their clinical utility is limited by an acute inflammatory response post‐administration. LNP‐associated inflammation (LAI) manifests as *de novo* inflammation in select organs (e.g., in the lungs after inhalation) and severely worsens pre‐existing inflammation. NF‐κB is a key transcription factor mediating this inflammation and represents a promising target for LAI mitigation strategies. However, systemic NF‐κB inhibition carries risks of global immunosuppression, highlighting the need for localized inhibition. Here, we screen a panel of direct and indirect small molecule inhibitors of NF‐κB for their ability to reduce LAI without disrupting LNPs’ expression. We identify inhibitors that load efficiently into LNPs, retain anti‐inflammatory activity in vitro and in vivo, and preserve expression of cargo mRNA in target tissues. Our results demonstrate that co‐formulation of NF‐κB inhibitors with cargo nucleic acids enables localized immunomodulation in LNPs, offering a modular strategy to enhance the safety profile of LNPs for therapeutic use in inflammatory or high‐risk patient populations.

## Graphical abstract

Lipid nanoparticles (LNPs) activate NF‐κB and provoke inflammation that limits therapeutic delivery. By encapsulating selective NF‐κB inhibitors within LNPs, localized suppression of inflammatory signaling is achieved without compromising mRNA expression. Enhanced formulations improve drug retention and reduce cytokine responses in vivo, providing a simple and modular approach to engineering better‐tolerated LNP therapies.

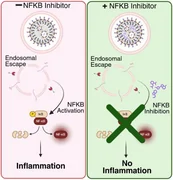

## 1 Introduction

Lipid nanoparticles (LNPs) are a cornerstone of modern nucleic acid therapeutics, enabling safe and efficient delivery of cargo such as mRNA, siRNA, and plasmid DNA to target cells [1, 2, 3, 4]. Their clinical utility has been validated in diverse applications including vaccines, gene editing, and protein replacement therapy. However, despite their transformative potential, a persistent limitation of LNP‐based platforms is their tendency to induce inflammation upon administration [5, 6]. This lipid nanoparticle‐associated inflammation (LAI) occurs across delivery routes—including intravenous, intradermal, and intratracheal—and can be driven by innate immune sensing of the nanoparticle itself or by secondary effects, such as endosomal disruption during cargo release [5].

While LAI is generally tolerated in healthy individuals, it is significant enough that several clinical trials have been halted [7, 8]. In addition, LNP‐triggered inflammation can exacerbate pre‐existing pathology, increase systemic cytokine levels, and limit therapeutic efficacy [5, 6]. Therefore, strategies that reduce or localize LNP‐induced inflammation are urgently needed to expand the applicability of LNPs beyond healthy populations.

Arguably the most central and critical player in the inflammatory cascade is the nuclear factor kappa‐light‐chain‐enhancer of activated B cells (NF‐κB), a master transcription factor regulating the expression of pro‐inflammatory cytokines such as IL‐6, TNFα, and IFN‐β [9, 10, 11]. NF‐κB has been repeatedly implicated as a major effector in LNP‐induced inflammation [5, 12, 13, 14]. Once activated—typically via IκBα phosphorylation and degradation—NF‐κB translocates to the nucleus, where it drives transcription of innate immune genes [10]. Therefore, direct inhibition of the NF‐κB pathway presents an attractive strategy for mitigating LAI.

There are three primary mechanisms by which NF‐κB can be pharmacologically inhibited: (1) prevent phosphorylation of IκBα by blocking IκB kinase (IKK) activity or the NEMO domain; (2) inhibiting proteasomal degradation of phosphorylated IκBα to retain NF‐κB in the cytoplasm; and (3) directly preventing nuclear translocation of activated NF‐κB dimers [15, 16]. In addition to pharmacological methods, siRNA targeting of the p65 subunit of NF‐κB offers a gene‐silencing approach [17, 18]. However, due to the constitutive expression and stability of NF‐κB proteins, siRNA knockdown requires extended pre‐treatment and is not well‐suited to mitigate the acute inflammatory response elicited by LNPs. LAI is initiated within hours of administration and is primarily driven by innate immune cells, particularly macrophages, due to their robust capacity for foreign particle clearance and cytokine production [5, 6]. Thus, small molecule inhibitors offer a more practical, fast‐acting alternative for transient NF‐κB inhibition in the context of LNP administration.

However, systemic inhibition of NF‐κB carries significant risk, particularly in immunocompromised or critically ill patients [19]. Broad suppression of NF‐κB can lead to pan‐immunosuppression, increasing vulnerability to infection and/or impairing necessary inflammatory responses [20, 21]. To address this, we propose a strategy in which NF‐κB inhibitors are encapsulated directly into LNPs. This approach enables spatially and temporally confined delivery of anti‐inflammatory agents, targeting inflammation specifically at the site of LNP uptake and reducing off‐target effects on systemic immunity [22].

We hypothesize that the inclusion of a small molecule NF‐κB inhibitor within the LNP formulation will suppress LAI locally, without compromising delivery efficacy or expression of the nucleic acid cargo. In this study, we systematically screen a panel of direct and indirect NF‐κB inhibitors to: Identify compounds that attenuate LAI; Evaluate their encapsulation efficiency and stability within LNPs; and validate their ability to function in vivo without impairing mRNA expression.

Through this work, we aim to establish a rational framework for engineering anti‐inflammatory LNPs with the potential to enhance safety and therapeutic reach across a broader range of patient populations.

## 2 Results

### 2.1 LAI is Mediated by NF‐κB Activation

**FIGURE 1:**
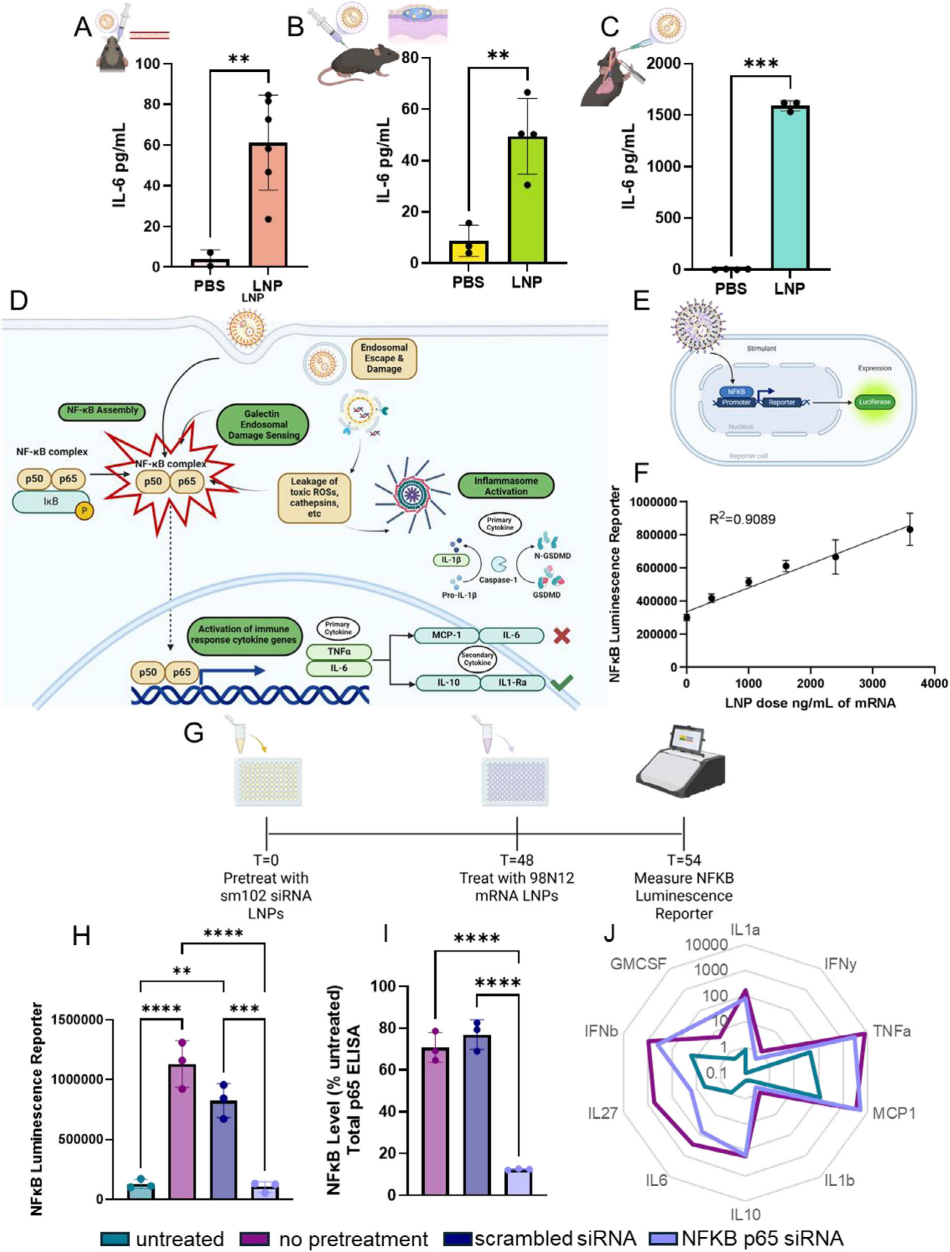
NF‐κB is activated by LAI and NF‐κB activity is required for expression of most inflammatory cytokines involved in LAI. LNPs induce inflammation in naive mice across a variety of administration routes. (A) SM‐102 LNPs delivered intravenously at a dose of 10 µg RNA, (B) SM‐102 LNPs delivered intradermally at 1 µg, and (C) SM‐102 LNPs delivered intratracheally at a dose of 7.5 µg all have significant increases in IL‐6 production in plasma, tissue homogenate, and BAL respectively. (D) Graphical representation of LAI highlighting the central role of NF‐κB in the response. (E) NF‐κB Luciferase Reporter line where activation of NF‐κB in RAW 264.7 Macrophages leads to production of luciferase. (F) NF‐κB Luciferase reporter cells showed a dose‐dependent increase in NF‐κB activity when treated with LNPs. (G‐H) NF‐κB activity after 1000 ng/mL 98N12 LNP treatment was significantly decreased in cells pretreated with 400 ng/mL NF‐κB p65 siRNA SM‐102 LNPs compared to those with no pretreatment or pretreated with scrambled siRNA LNPs. (I) Decreased NF‐κB activity aligned with decrease in NF‐κB p65 levels, relative to the untreated group, as measured via NF‐κB p65 ELISA. (J) Inhibition of NF‐κB activity via NF‐κB p65 siRNA also led to a decrease in cytokines of 98N12 LNP treated cells compared to cells with no pretreatment. Statistics: For (A, B, and C) *n* = 6, 4, and 4 biological replicates respectively; (C) had two technical replicates for each biological replicate. For (F and H–J), *n* = 3 and data shown represents mean ± SEM. (A–C) and (H,I), comparisons were made using one‐way ANOVA with Tukey's post‐hoc test. ^**^ = *p* <0.01, ^***^ = *p* <0.001, ^****^ = *p* <0.0001 Created in BioRender. Brenner, J. (2026) https://BioRender.com/4jf9ur8.

LNPs are routinely used across diverse administration routes; however, they elicit inflammatory responses even under non‐pathological conditions [5, 23, 24, 25]. Here we demonstrate LAI across a variety of administration routes in vivo. Intravenous, intradermal, and intratracheal administration of LNPs all resulted in statistically significant increase of IL‐6 production compared to vehicle treated mice indicating inflammation caused by the delivered LNPs (Figure 1A–C). From previous work in our lab, we established LAI is primarily induced through endosomal damage during nucleic acid cargo escape [5]. In addition to endosomal damage sensing, the leak of endosomal contents such as cathepsins and ROS also propagate an inflammatory response [5].

Given the central role of NF‐κB in mediating inflammatory responses, we hypothesized that NF‐κB activation is a key downstream effector in LAI [9, 10, 11, 15]. A schematic overview of this pathway is depicted in Figure 1D. To directly assess NF‐κB activity, we studied a NF‐κB reporter cell line where an increase in NF‐κB activity results in increased luciferase expression and subsequent luminescence (Figure 1E). The RAW 264.7 murine macrophage‐like cell line was selected due to macrophages’ critical role in inflammatory responses and our previous work showing good homology with in vivo results [6, 15]. Upon treatment with increasing doses of LNPs, we observed a dose‐dependent increase in luminescence, indicating NF‐κB activation (Figure 1F).

To confirm the necessity of NF‐κB in mediating the LAI, we used siRNA to knock down the p65 subunit of NF‐κB. Given the prolonged half‐life of inactive NF‐κB, cells were pretreated with p65‐targeting siRNA using less‐inflammatory LNPs [16]. Forty‐eight hours later, cells were treated with a second, pro‐inflammatory LNP formulation (Figure 1G). NF‐κB activity, as measured by the luciferase reporter line, was significantly attenuated in p65 siRNA‐treated cells compared to untreated or scrambled siRNA controls, reaching NF‐κB activity levels equal to that of naive cells (Figure 1H). ELISA confirmed a corresponding decrease in p65 protein levels, with p65 siRNA‐treated cells exhibiting <20% of p65 levels compared to control groups (Figure 1I). Furthermore, pretreatment of cells with NF‐κB p65 siRNA resulted >2‐fold reduction in key cytokines after subsequent LNP treatment in comparison to cells with no pretreatment including TNFα, IL‐6, IFN‐β (Figure 1J). Together, these results support the importance of NF‐κB in LAI.

### 2.2 Screening Small Molecule Inhibitors of NF‐κB to Mitigate LAI

**FIGURE 2:**
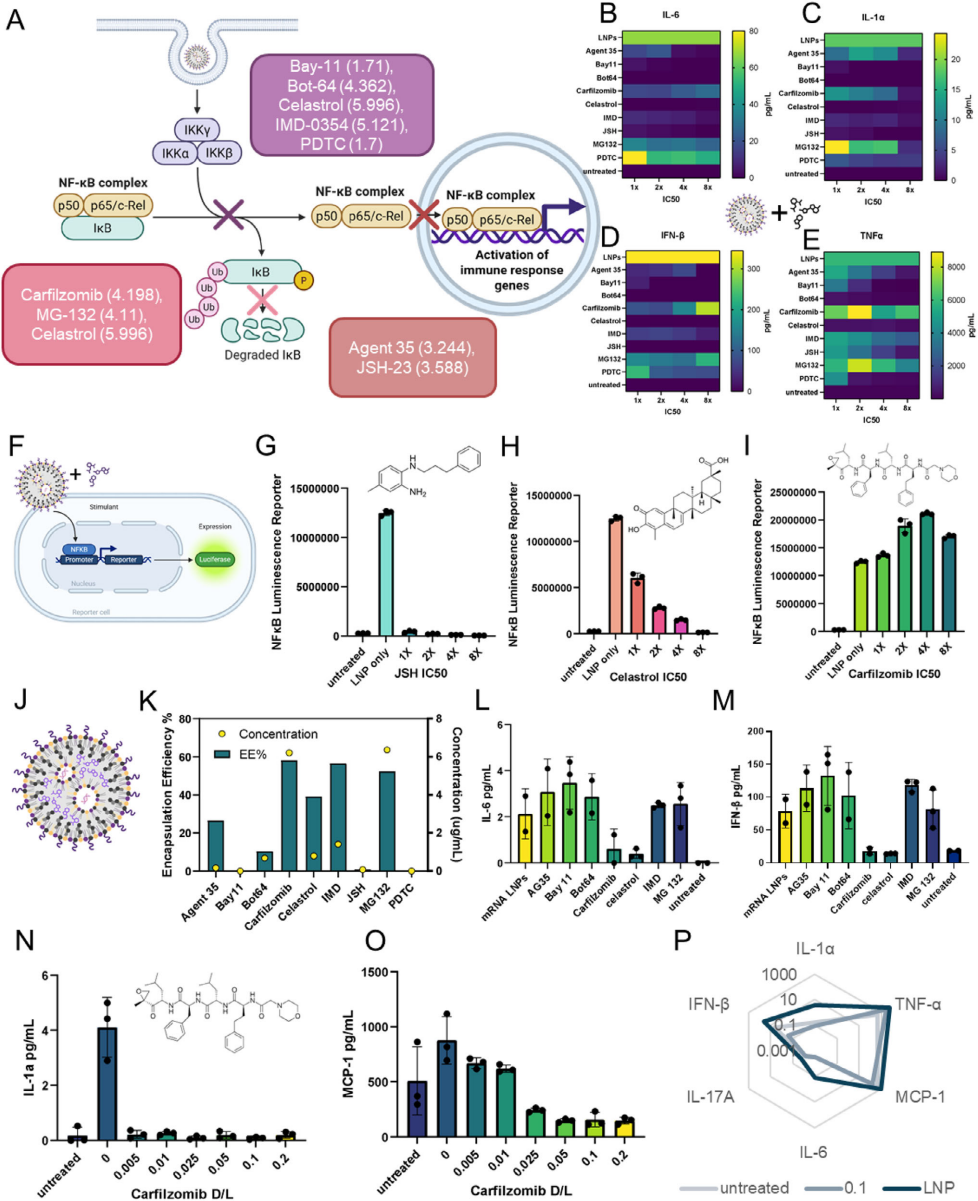
Small Molecule Drug screening shows all tested NF‐κB inhibitors dimmish LAI up to 100% reduction. (A) Graphic represents the various methods of NF‐κB inhibition including both direct and indirect methods. Direct methods include inhibition of the NEMO complex (purple) and inhibition of nuclear localization (red). Indirect methods include inhibition of IκBα degradation through inhibition of the proteasome (pink). Drugs seen here were screened through literature and selected due to high logP values (in parentheses). (B–E) Co‐treatment of free small molecule inhibitors (1‐8X IC50) and 98N12 LNPs (dose = 400 ng/mL) to macrophages in vitro saw dose dependent decreases in cytokine production for key inhibitors. Celastrol and JSH‐23 showed reductions in IL‐6 (B), IL‐1α (C), IFN‐β (D), and TNFα (E). (F) More direct measurement of NF‐κB activity was done by co‐treating NF‐κB luciferase reporter line with the small molecule inhibitors and 98N12 LNPs (dose = 1000 ng/mL). (G) JSH‐23 reduced NF‐κB activity back to baseline at its reported IC50 dose. (H) Celastrol showed a clear dose response decrease on NF‐κB activity with reduction back to baseline activity at 8X IC50. (I) Treatment of reporter cells with free carfilzomib showed no difference or an increase in NF‐κB activity. (J) LNPs were then formulated with the small molecule inhibitors in the lipid (organic) phase of the formulation. (K) Drug concentration and encapsulation efficiency as measured via UPLC. LNPs formulated with small molecule drugs only saw decreases in (L) IL‐6 and (M) IFN‐β levels in vitro for celastrol and carfilzomib. Carfilzomib was loaded into LNPs at doses ranging from 0.005 to 0.2 D/L. (N‐O) Dose response of carfilzomib formulated LNPs IL‐1α and MCP‐1. (P) Additional cytokines show a reduction for 0.1 D/L carfilzomib 98N12 LNPs. Statistics: For (B‐E) and (P), *n* = 3 and the data is shown represents mean. For (L‐M), *n* = 2 or 3 and the data shown represents mean ± SEM. All other graphs, *n* = 3 and the data shown represents mean ± SEM. Created in BioRender. Brenner, J. (2026) https://BioRender.com/4jf9ur8.

Due to NF‐κB's crucial role in LAI, we sought to find quick acting inhibitors to prevent an inflammatory response after LNP administration. Ten candidate inhibitors were selected based on literature precedence, mechanism of action (e.g., inhibition of IκBα phosphorylation, degradation, or NF‐κB nuclear translocation), and hydrophobicity (LogP values) (Figure 2A) [26, 27, 28, 29, 30, 31, 32, 33].

Initial screening involved co‐treatment of RAW macrophages with LNPs and free small molecule inhibitors. Cytokine analysis revealed that most compounds reduced pro‐inflammatory cytokine secretion by varying degrees, but JSH‐23 and celastrol consistently reduced IL‐6, IL‐1α, IFN‐β, and TNFα back to baseline without compromising cell viability or mRNA cargo expression (Figure 2B–E; Figure S1). This is similarly observed in differentiated human THP‐1 cells indicating conservation of JSH‐23 and celastrol efficacy across species (Figure S5D–I).

We next evaluated the direct effect of these inhibitors on NF‐κB activity using the NF‐κB luciferase reporter cell line (Figure 2F). JSH‐23 completely reduced NF‐κB activity back to baseline at its reported IC50, while celastrol exhibited a clear dose‐response effect on NF‐κB activity (Figure 2G,H) [31, 34]. In contrast, carfilzomib, a proteasome inhibitor, showed either no change in NF‐κB activity or a slight increase at higher doses (Figure 2I).

To assess encapsulation potential, each small molecule drug was incorporated into the lipid phase of the LNP during formulation without changing lipid component ratios (Figure 2J). Drug encapsulation efficiency was measured via UPLC comparing samples pre‐ and post‐removal of free drug via Zeba desalting column. Of the drugs tested carfilzomib, celastrol, IMD‐0354, and MG‐132 had an encapsulation efficiency ≥40% (Figure 2K). Functionally, only carfilzomib‐ and celastrol‐loaded LNPs produced a robust reduction in IL‐6 and IFN‐β, achieving average cytokine decreases of approximately four‐ and five‐fold, respectively (Figure 2L,M). It is of note that carfilzomib when treated as a free inhibitor has no or slightly inflammatory effect on cytokine production and NF‐κB activity while LNP encapsulated carfilzomib shows a strong reduction in cytokines (Figure 2B–E, I, L–P). Dose‐response experiments confirmed strong cytokine suppression for carfilzomib across a range of drug‐to‐lipid (D/L) molar ratios with ∼20‐fold and 6‐fold decrease in IL‐1α and MCP‐1 respectively at 0.2 D/L (Figure 2N–P). Importantly, carfilzomib loading did not compromise cell viability or RNA cargo expression (Figure S2).

### 2.3 Rational Formulation of Celastrol‐LNPs for NF‐κB Suppression

**FIGURE 3:**
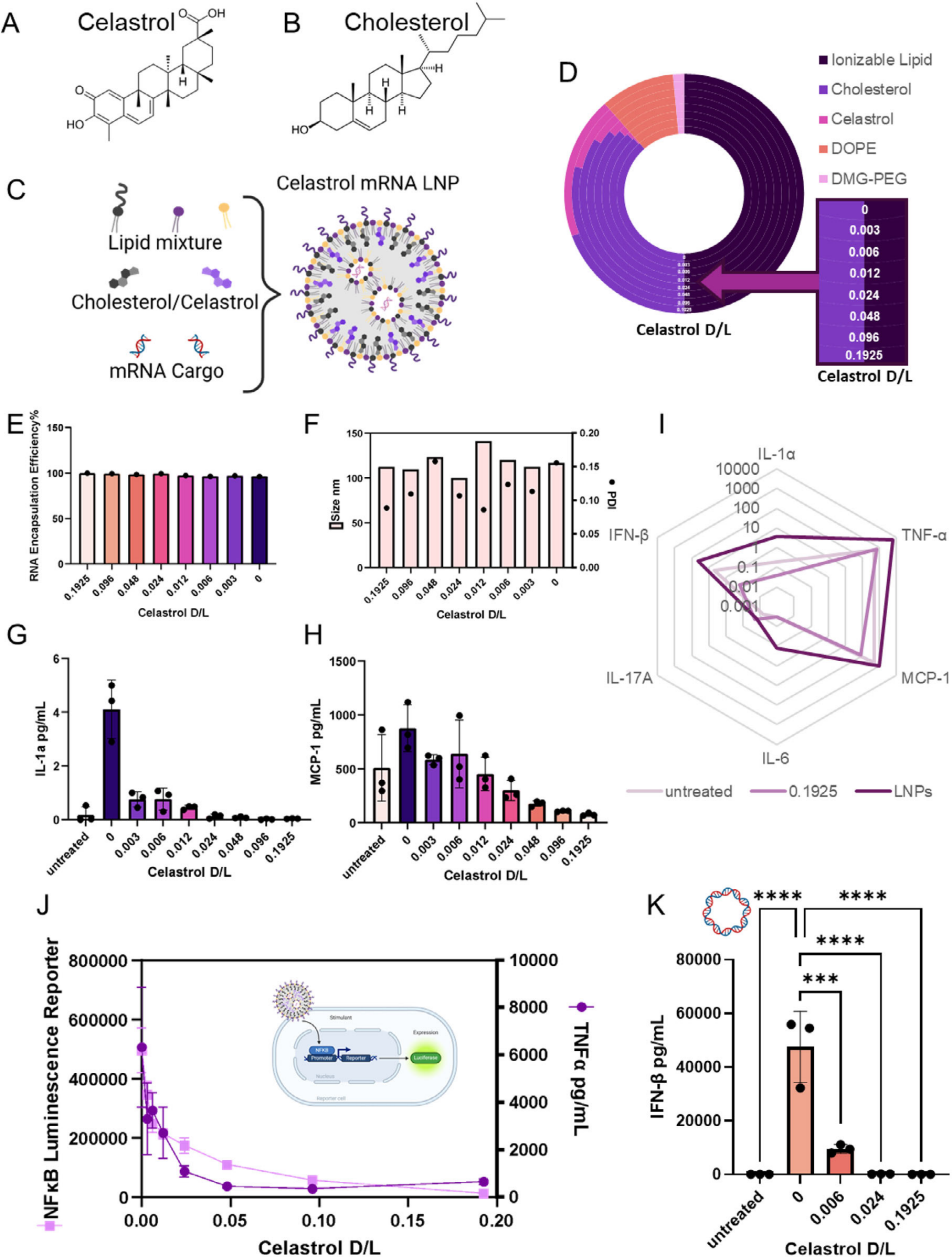
Celastrol's similarity to cholesterol offers high‐capacity loading into standard LNPs. (A) Structure of celastrol shows strong homology to (B) the structure of cholesterol, a common component in LNP formulation. (C) Graphic showing LNP formulation where cholesterol is replaced by celastrol. (D) LNP formulation lipid percentages with celastrol replacement of cholesterol. Quality controls of celastrol LNPs saw (E) excellent RNA encapsulation and (F) consistent size and PDI. (G,H) Dose response of celastrol formulated LNPs IL‐1α and MCP‐1. (I) Additional cytokines show reduction for 0.1925 D/L celastrol 98N12 LNPs. (J) Treatment of NF‐κB Luciferase reporter RAW Macrophages with celastrol 98N12 LNPs, at a dose of 1000 ng/mL of mCherry mRNA, saw a clear dose response decrease of NF‐κB activity as measured by luminescence (left). This followed very closely with TNFα production levels in the cell supernatant (right). (K) Celastrol significantly decreased IFN‐β levels due to DNA associated inflammation. Statistics: For (I), *n* = 3 and the data shown represents mean. (G,H) and (J,K), *n* = 3 and the data shown represents mean ± SEM. All comparisons were made using one‐way ANOVA with Tukey's post‐hoc test. ^***^ = *p* <0.001, ^****^ = *p* <0.0001 Created in BioRender. Brenner, J. (2026) https://BioRender.com/4jf9ur8.

Celastrol, a triterpenoid structurally similar to cholesterol, was used to partially replace cholesterol in the LNP formulation (Figure 3A–C). Formulations tested included celastrol substitution from 0.3% to 19.25% of total lipid content (Figure 3D). 19.25% total lipid content represents a 50% substitution of cholesterol with celastrol. Irrespective of celastrol percentage, LNPs formed were similar sizes (116.87 ± 4.26) and had similar polydispersity indexes (PDI) (0.1179 ± 0.00958) (Figure 3F). Additionally, increasing celastrol did not impact nucleic acid cargo encapsulation (Figure 3E). Both mRNA encapsulation efficiency and PDI checks passed our QC requirements indicating stable particles suitable for downstream applications.

Celastrol‐loaded LNPs showed potent and dose‐dependent cytokine suppression as cholesterol replacement increased, with the highest encapsulated dose producing approximately a 100‐fold reduction in IL‑1α and a 10‐fold reduction in MCP‑1 (Figure 3G–I). Importantly, neither inhibitor compromised cell viability or RNA cargo expression, except at celastrol's highest dose where viability and expression decreased ∼10% (Figure S2).

In the NF‑κB luciferase reporter cell line, celastrol‐loaded LNPs suppressed NF‑κB activation in a dose‐dependent manner, correlating with TNFα levels in the cell supernatant (Figure 3J). The calculated IC50 values were 0.0084 D/L for TNFα and 0.0073 D/L for NF‑κB activity as measured by luminescence. Celastrol also suppressed DNA‐induced inflammation – driven primarily by cGas‐STING pathway – indicating its broader immunosuppressive potential beyond LAI (Figure 3K) [4, 35, 36].

It is of interest to note the difference of NF‐κB inhibition between free vs loaded small molecule drug. Free celastrol reduces NF‐κB activity by ∼50% at 0.2 µm in the NF‐κB luciferase reporter line (Figure 1H). Meanwhile, ∼50% reduction is achieved with 0.006 D/L celastrol‐LNPs (Figure 3J). To achieve the same inhibitory effect, ∼2E‐11 moles of free celastrol are required, while ∼8e‐15 moles of encapsulated celastrol are required, indicating a ∼2500‐fold difference in celastrol to induce the same inhibitory effect. These results further support the importance of inhibitor‐LNP encapsulation, in addition to preventing pan immunosuppression.

### 2.4 Improved Celastrol Retention via Two‐Step Mixing Formulation

**FIGURE 4:**
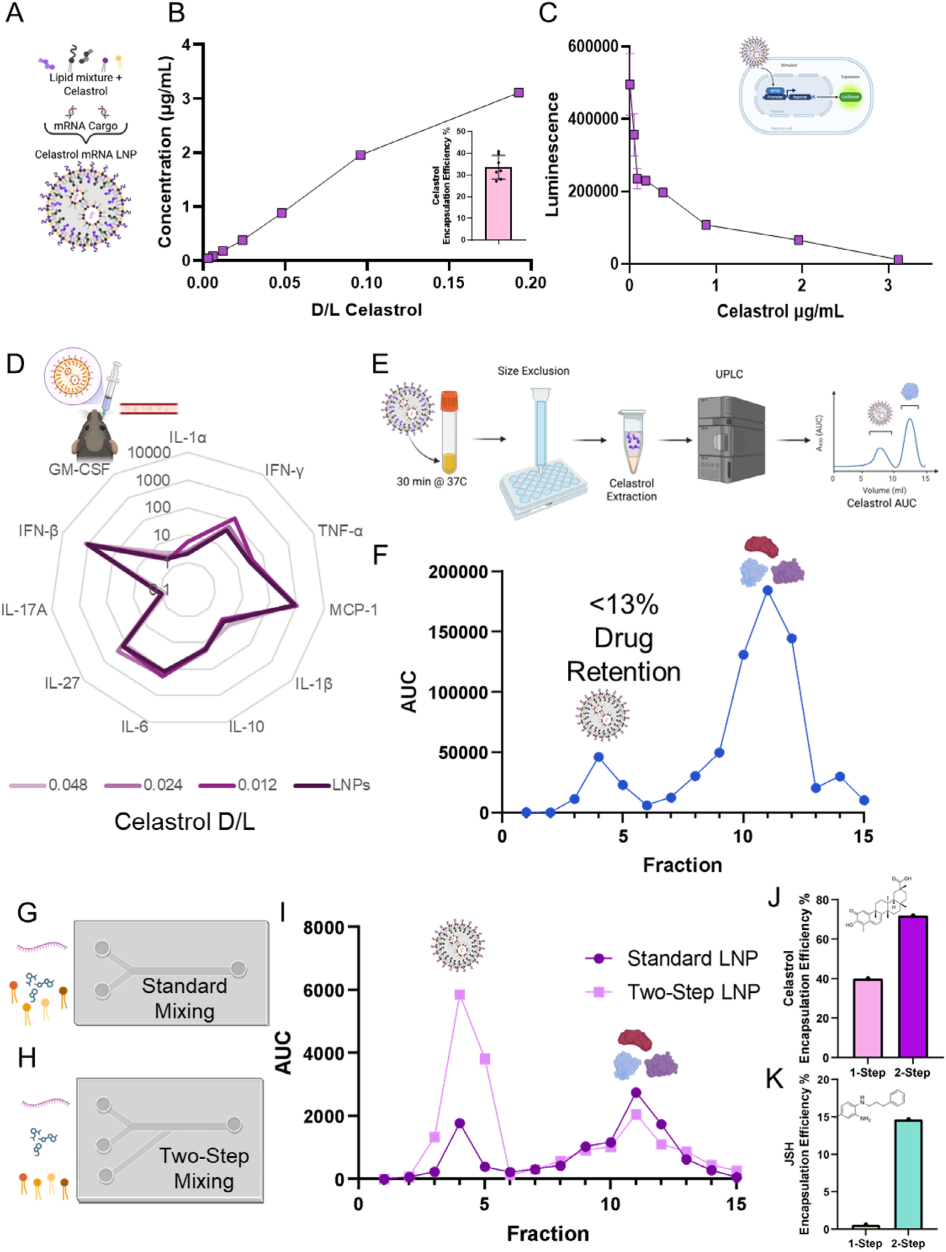
Celastrol rapidly leaks out of standard LNPs in vivo but can be retained in LNPs when synthesized in a 2‐step mixing process. (A) Celastrol LNPs were formulated through cholesterol replacement with celastrol in the lipid phase. (B) Drug encapsulation efficiency was similar regardless of the amount of celastrol added during formulation. (C) Clear dose dependent NF‐κB activity inhibition was seen in the NF‐κB luminescence reporter line when treated with celastrol‐LNPs (dose = 1000 ng/mL RNA 98N12 LNP). (D) In vivo delivery of celastrol LNPs at 10 µg dose saw no difference in inflammation as measured by plasma cytokines when delivered intravenously. (E) Schematic of testing ex vivo celastrol leak. Celastrol loaded LNPs were incubated with naive plasma for 30 min at 37C. The sample then went through size exclusion to separate LNPs from plasma protein. Celastrol was then extracted from each fraction and ran on UPLC to measure amount remaining in the LNPs vs. adsorbed to the proteins. Standard preparation of celastrol LNPs, shown in Figure 4G, results in less than 13% drug retention in the LNPs (F). A new two‐step mixing method for LNP formulation, shown in Figure 4H, lead to an increase in celastrol retention after plasma incubation (I). The two‐step mixing method increased the encapsulation efficiency of celastrol (J) and allowed for JSH‐23 encapsulation (K). Statistics: For (B) inset, *n* = 7 and the data shown represents mean ± SEM. For (C), *n* = 3 and the data shown represents mean ± SEM. For (D), *n* = 3 and the data shown represents mean. Created in BioRender. Brenner, J. (2026) https://BioRender.com/4jf9ur8.

In order to optimize and further characterize celastrol LNP formulation, we looked at celastrol loading via cholesterol replacement in the lipid phase (Figure 4A). This loading method resulted in drug encapsulation sitting just below 35% (Figure 4B). Functionally, there was a clear dose dependent response to reduce NF‐κB activity as measured via NF‐κB luciferase reporter line (Figure 4C).

Despite significant reduction of LNP‐induced cytokine production in vitro, celastrol‐loaded LNPs saw no difference in cytokine production when administered intravenously with different celastrol doses (Figure 4D). Given celastrol's strong structural similarity to cholesterol, we hypothesized celastrol could be leaking from the LNPs in vivo due to exchange with circulating lipoproteins [37, 38]. To test this, we designed a method to see celastrol retention in LNPs after incubation with plasma using a previously described extraction method [39]. After incubating LNPs in plasma at 37°C, simulating LNPs after IV injection, we used size exclusion to separate LNPs from plasma proteins (Figure 4E). Celastrol was then extracted from each fraction using tertbutyl ether and analyzed via UPLC to determine the amount of drug remaining in LNPs vs. adsorbed to protein. We saw <13% of drug retained in the LNPs after 30 min of incubation, supporting the hypothesis that celastrol is quickly leaking out of the LNPs when injected intravenously (Figure 4F).

To improve retention, we altered the LNP formulation method. Previously, we had two portions we mixed together for LNPs: 1. Nucleic acid cargo in aqueous buffer 2. Lipid and small molecule drug organic mixture (Figure 4G). The new formulation includes three portions mixed in two steps: 1. Nucleic acid cargo in aqueous buffer 2. Small molecule drug in organic mixture 3. LNP lipids in an organic mixture. Portions 1 and 2 are mixed together and subsequently mixed with 3 (Figure 4H). The hypothesis is that two‐step mixing will increase the amount of small molecule drug encapsulated in the nanoparticle and decrease drug leak in plasma. Two‐step mixing resulted in doubled drug retention than standard mixing after plasma incubation and nearly doubled celastrol encapsulation efficiency (Figure 4I,J). Additionally, the improved method enabled encapsulation of JSH‐23, which had previously shown 0% loading using conventional approaches (Figure 4K). This effect is very notable as several other loading strategies failed for JSH‐23, including active loading and pro‐drug approaches (Figure S3).

### 2.5 In Vivo Evaluation of NF‐κB‐Inhibitor Loaded LNPs

**FIGURE 5:**
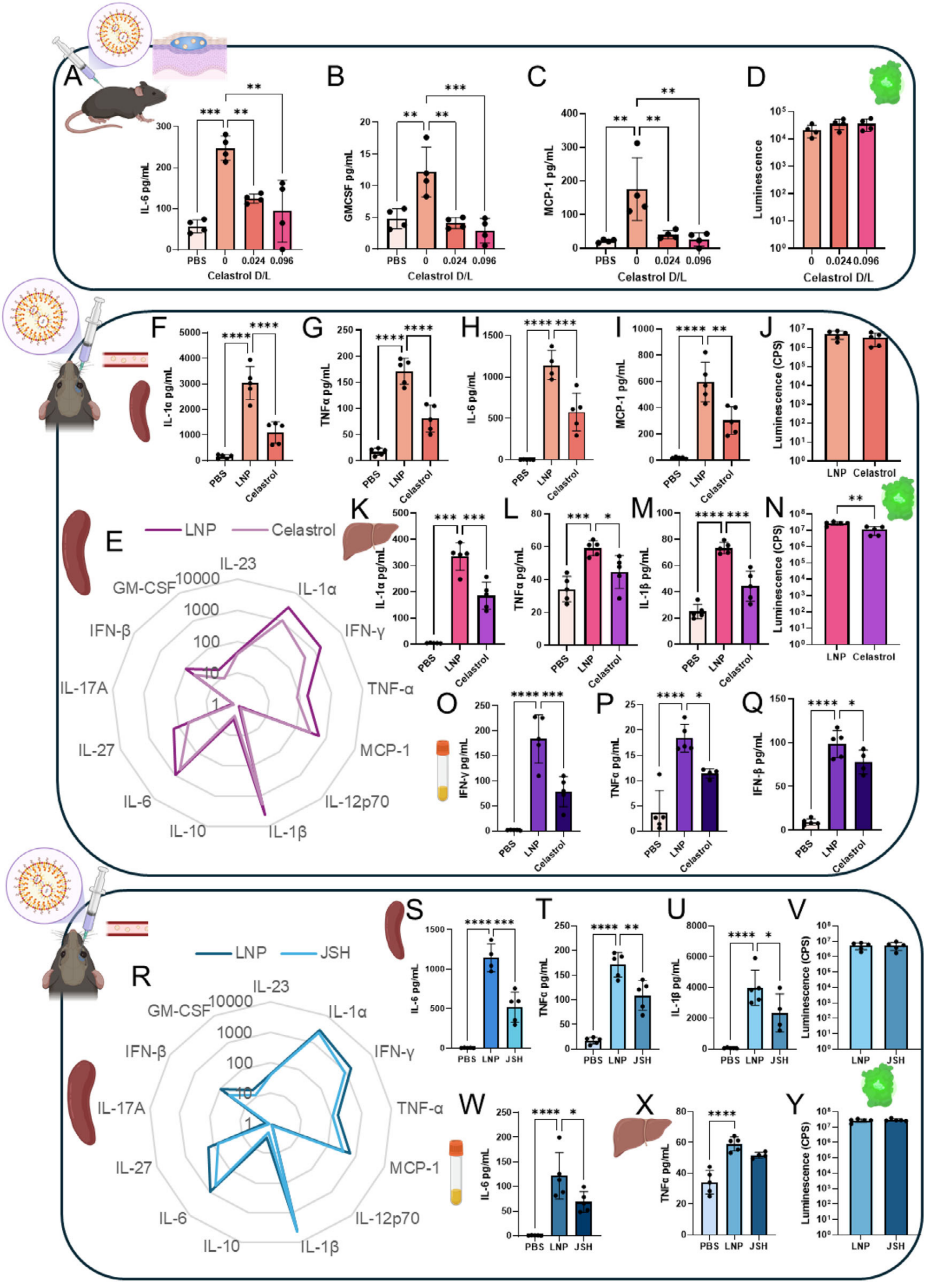
mRNA‐LNPs co‐loaded with NFKB inhibitors show significant reductions in key cytokines in vivo. (A–D) 98N12‐5 LNPs containing celastrol at two different concentrations were delivered intradermally at a dose of 1 µg mRNA. (A) IL‐6, (B) GMCSF, and (C) MCP‐1 all saw significant reduction in cytokine levels in celastrol containing LNPs compared to LNPs without celastrol. (D) Saw no change in expression of mRNA cargo with celastrol LNPs. (E–Q) Two‐step mixing SM‐102 LNPs with or without celastrol were delivered intravenously at a dose of ∼10 µg (0.4 mg/kg). (E) Spleen homogenate cytokine levels decreased in celastrol LNP treated mice compared to LNP mice. (F–J) With significant decrease in key cytokines including IL‐1α, TNFα, IL‐6, and MCP‐1 while maintaining mRNA expression as measured by luminescence. (K–M) Similarly, liver homogenate of celastrol LNP treated mice showed decreased cytokine levels including IL‐1α, TNFα, and IL‐1β. (N) Liver homogenate showed ∼2‐fold lower mRNA expression with both formulations expressing in the 10^7^ range. (O‐Q) Overall plasma cytokines were also lower in celastrol treated mice with statistical significance in IFN‐γ, IFN‐β, and TNFα. (R‐V) JSH‐23 loaded LNPs saw decrease in spleen homogenate cytokines with no statistically significant change in mRNA expression. (W) IL‐6 levels were significantly lower in plasma of JSH‐23 LNP treated mice. (X,Y) JSH‐23 slightly lowered TNFα in liver homogenate with no decrease in mRNA cargo expression. Statistics: For (A–D), *n* = 4 and the data shown represents mean ± SEM. For (E) and (R), *n* = 5 biological replicates; the data shown represents mean. (F–Q) and (S–Y), *n* = 5 biological replicates; the data shown represents mean ± SEM. (J), (N), (V), and (Y) comparisons were made using an unpaired *t*‐test with Welch's correction. All other comparisons were made using one‐way ANOVA with Tukey's post‐hoc test. ^*^ = *p*<0.05, ^**^ = *p* <0.01, ^***^ = *p* <0.001, ^****^ = *p* <0.0001 Created in BioRender. Brenner, J. (2026) https://BioRender.com/4jf9ur8.

To evaluate therapeutic efficacy in vivo, we first tested celastrol‐loaded LNPs via intradermal injection. This route induces localized inflammation and minimizes systemic circulation, reducing the impact of drug leak [40, 41]. Celastrol‐LNPs significantly reduced IL‐6, GMCSF, and MCP‐1 levels in comparison to skin injected with LNPs without celastrol (Figure 5A–C). Both GMCSF and MCP‐1 were reduced back to that of the PBS vehicle group and IL‐6 saw ∼50% reduction for both doses of celastrol. Additionally, mRNA expression was unaffected by celastrol inclusion in LNPs (Figure 5D).

Following validation of the two‐step mixing method, celastrol‐LNPs were administered intravenously. Untargeted LNPs primarily end up in the liver and spleen, which we have previously shown both through nucleic acid cargo expression and radiotracing of nanoparticles (Figure S8) [42, 43]. Therefore, we collected the liver and spleen to see organ specific inflammation and mRNA expression in addition to collecting plasma for systemic inflammation. Compared to control LNPs, celastrol‐LNPs broadly reduced cytokine levels in spleen homogenate, liver homogenate, and plasma (Figure 5E–Q). Cytokine profiling revealed significant reduction in IL‐1α and TNFα in both liver and spleen homogenate and significant reduction in IFNs and TNFα in the plasma (Figure 5F,G,K,L,O–Q). mRNA expression was unchanged in the spleen and approximately two‐fold reduction in the liver indicating mRNA delivery and translation are undisrupted (Figure 5J,N).

JSH‐23‐loaded LNPs also significantly decreased cytokine levels in spleen homogenate and plasma, though their anti‐inflammatory efficacy in the liver was limited (Figure 5R–Y). These results may reflect the more selective function of JSH‐23 compared to celastrol, which may modulate additional inflammatory pathways. JSH‐23 had no statistically significant impact on expression of mRNA cargo (Figure 5V,Y).

## 3 Discussion

LNPs have revolutionized nucleic acid delivery, but their inherent immunogenicity remains a significant barrier to repeated or systemic dosing [5, 6]. Here, we identify the transcription factor NF‐κB as a central driver of LAI and present a strategy to mitigate this effect by co‐delivering small molecule NF‐κB inhibitors via modified LNP formulations.

Our data demonstrate that LNPs, independent of administration route, induce a robust pro‐inflammatory response characterized by elevated cytokine production (IL‐6, TNFα, IFN‐β, etc.), which is tightly coupled to NF‐κB activation. Using an NF‐κB luciferase reporter system and targeted knockdown of the p65 subunit, we establish NF‐κB as both necessary and sufficient for this inflammatory response. Importantly, this mechanism appears to be downstream of endosomal damage and cathepsin/ROS release, consistent with known NF‐κB activation pathways following intracellular danger signaling [10].

Given the rapid onset of LAI following LNP administration and the limitation of slow kinetics in siRNA‐mediated NF‐κB knockdown, we explored pharmacological inhibition as a strategy to suppress NF‐κB signaling. Through literature‐guided screening, we identified celastrol and JSH‐23 as potent NF‐κB inhibitors capable of restoring inflammatory cytokine levels to baseline without compromising cell viability or nucleic acid expression. Celastrol, in particular, emerged as a dual‐functional compound—both a pharmacologic inhibitor of NF‐κB and a structural analog of cholesterol, enabling facile incorporation into LNPs through partial cholesterol substitution. When delivered intradermally, celastrol‐loaded LNPs reduced key pro‐inflammatory cytokines (IL‐6, GM‐CSF, MCP‐1) without abolishing transgene expression, highlighting the advantage of local administration for mitigating drug leakage while maintaining therapeutic delivery.

However, intravenous administration of celastrol‐loaded LNPs failed to suppress systemic inflammation, prompting an investigation into drug retention. We show that celastrol rapidly leaks from LNPs upon plasma exposure—an effect likely driven by its cholesterol mimicry and exchange with lipoproteins [37, 38]. By employing a two‐step formulation strategy that physically separates celastrol from the primary lipid phase until after nucleic acid complexation, we achieved markedly higher encapsulation efficiencies and reduced drug leakage. This reformulation not only preserved celastrol's anti‐inflammatory efficacy but also enabled successful encapsulation of JSH‐23, previously incompatible with LNP loading.

The two‐step formulation produced significant in vivo benefits with both NF‐κB inhibitors. Upon intravenous administration, the improved retention of celastrol in LNPs translated to systemic suppression of pro‐inflammatory cytokines in plasma, liver, and spleen [42, 43]. Importantly, these effects occurred without substantial loss of mRNA expression in the spleen and ∼2‐fold change in the liver, suggesting that co‐delivery of NF‐κB inhibitors does not compromise therapeutic efficacy.

JSH‐23‐loaded LNPs showed similar trends, though with slightly reduced efficacy, particularly in hepatic tissues. This may be due to JSH‐23's distinct specificity for NF‐κB inhibition or more limited bioactivity compared to celastrol, which has additional effects beyond NF‐κB blockade, including heat shock protein modulation and redox regulation [26, 44, 45, 46]. Nonetheless, JSH‐loaded LNPs significantly decreased splenic inflammation, reinforcing the potential of this strategy across structurally and mechanistically diverse inhibitors.

While NF‐κB is a central and well‐characterized regulator of inflammation, it is not the sole transcriptional mediator of inflammatory responses. Inflammatory signaling is governed by a network of transcription factors—including IRFs, STATs, and AP‐1—each contributing to the overall cytokine production [12, 47]. The strategies developed in this study, including siRNA, aptamer, peptide, and small molecule‐based inhibition, as well as advances in LNP formulation, provide a versatile framework for targeting other pro‐inflammatory pathways beyond NF‐κB.

A key insight from our work is the extent and rapidity of small molecule drug leak from conventional LNP formulations. We demonstrate that modifying the formulation process can significantly reduce drug loss, enabling more efficacious, inflammation‐suppressing LNPs. These advancements position us to expand this approach to other signaling axes involved in LNP‐induced inflammation and to rationally design multifunctional LNPs that modulate immunity with greater precision.

Additionally, our findings provide a more nuanced understanding of NF‐κB's role in LAI. While transient inhibition of NF‐κB was sufficient to suppress inflammation without compromising mRNA expression, it is important to recognize the potential risks of broad or sustained NF‐κB suppression. NF‐κB plays key roles in cell survival and immune homeostasis, and many NF‐κB inhibitors are used in oncology research specifically to induce apoptosis in cancer cells [48, 49, 50]. Notably, our data show that encapsulating NF‐κB inhibitors into LNPs allowed for equivalent suppression of NF‐κB activity in vitro using ∼2500‐fold lower doses compared to free drug co‐treatment. This targeted delivery could substantially reduce the risk of adverse effects associated with systemic NF‐κB inhibition.

It is also worth emphasizing that NF‐κB is not a single transcription factor, but rather a family of related dimers with diverse and sometimes opposing functions. The canonical pro‐inflammatory dimer, p65/p50, promotes cytokine production, while other dimers such as the p50/p50 homodimer can act as transcriptional repressors, promoting inflammation resolution [49, 51]. Most small molecule inhibitors likely do not discriminate between these NF‐κB complexes and may therefore suppress both pro‐ and anti‐inflammatory signaling. In contrast, siRNA‐mediated knockdown offers a subunit‐specific approach, such as selectively depleting p65, but is limited by its delayed kinetics and reliance on extended pre‐treatment. These considerations further support the need for rapid, localized, and tunable immunomodulation strategies—such as those described here—to safely control LNP‐induced inflammation.

In conclusion, we demonstrate that NF‐κB activation is a key mediator of LNP‐induced inflammation and can be effectively suppressed via co‐encapsulation of small molecule inhibitors. This approach enables the design of more tolerable and potentially more potent LNP‐based therapies. Our findings open a new avenue for the rational engineering of immune‐modulatory LNPs and may accelerate the development of next‐generation nanomedicines with reduced immunotoxicity and improved safety profiles.

## 4 Methods

### 4.1 Materials

DOPE (1,2‐dioleoyl‐sn‐glycero‐3‐phosphoethanolamine), cholesterol, DMG‐PEG 2000 (1,2‐dimyristoyl‐rac‐glycero‐3‐methoxypolyethylene glycol‐2000), AF594 (18:1 PE‐TopFluor AF594), and DSPC (1,2‐distearoyl‐sn‐glycero‐3‐phosphocholine) were purchased from Avanti Polar Lipids.

DOTAP (1,2‐Dioleoyl‐3‐trimethylammonium propane) was purchased from BroadPharm.

Ionizable lipid SM‐102 was purchased from Echelon Biosciences. 98‐N12‐5 ionizable lipid was purchased from MedChemExpress along with all drugs tested here with the exception of carfilzomib which was purchased from Cayman Chemical.

Details on use of these lipids in LNP formulations can be found in Figure S14.

5moU modified Luciferase mRNA and 5moU modified mCherry mRNA were purchased from Trilink. Luciferase plasmid DNA was purchased from Aldevron. NFKB p65 (RelA) siRNA was purchased from Horizon Discovery.

### 4.2 Animals

All experiments involving animals were conducted following the guidelines outlined in the Guide for the Care and Use of Laboratory Animals (National Institutes of Health, Bethesda, MD). Approval for all animal protocols was obtained from the University of Pennsylvania Institutional Animal Care and Use Committee [805696]. Male C57BL/6 mice aged 6–8 weeks (23‐25 g) sourced from The Jackson Laboratory, Bar Harbor, ME, were utilized for the experiments. Female BALB/cJ mice aged 7–8 weeks sourced from The Jackson Laboratory, Bar Harbor, ME, were utilized for In Vivo Imaging. The mice were housed in a controlled environment at temperatures between 22°C–26°C, adhering to a 12/12 h dark/light cycle, and had unrestricted access to food and water.

### 4.3 LNP Formulation

LNPs were formulated using the microfluidic mixing method. An organic phase containing a mixture of lipids dissolved in ethanol at a designated molar ratio (50:38.5:10:1.5, IL : Cholesterol : DOPE or DSPC : DMG‐PEG) was mixed with an aqueous phase (50 mM citrate buffer, pH 4) containing siRNA, mRNA or pDNA at a flow rate ratio of 1:3 and at a total lipid to mRNA weight:weight ratio of 40:1 in a microfluidic mixing device (NanoAssemblr Ignite, Precision Nanosystems). Two‐step was formulated following the same method as above with an additional organic phase containing small molecule inhibitors mixed at a flow rate ratio of 1:1:3 (aqueous: organic: organic). Further details on LNP formulation can be found in Figure S14. LNPs were dialyzed against 1× PBS in a 10 kDa molecular weight cut‐off cassette for 2 h and stored at 4°C. Further details regarding formulation and N:P ratios can be found in Figure S14.

### 4.4 LNP Characterization

Measurements of hydrodynamic nanoparticle size, distribution, polydispersity index, and zeta potential were conducted through dynamic light scattering using a Zetasizer Pro ZS from Malvern Panalytical. The encapsulation efficiencies and concentrations of LNP RNA or DNA were determined using a Quant‐iT RiboGreen RNA assay or Quant‐iT PicoGreen DNA assay (Invitrogen). Particle size and concentration were measured using Nanosight (Malvern Panalytical).

### 4.5 Drug Loading into LNPs

Small molecule drugs were added to the organic phase during LNP formulation as described above. In the case of two‐step mixing, drugs were in the first organic phase. Post dialysis, LNPs were passed through Zeba desalting column to remove unencapsulated drug. Samples prior to dialysis were collected and compared to post purification via UPLC to determine drug encapsulation concentration and efficiency.

### 4.6 Celastrol Extraction Post Serum Incubation

To test this, we designed a method to see celastrol retention in LNPs after incubation with plasma using a previously described extraction method [39]. After a 30‐min incubation at 37C of LNPs in mouse plasma, we used size exclusion to separate LNPs from plasma protein. Celastrol was then extracted from each fraction using tertbutyl ether. Samples were shaken ∼2000 rpm 5 min and spun down at 16 000 g for 5 min. Top portion was collected, evaporated, and resuspended in acetonitrile. Sample was run on UPLC to see the amount of drug remaining in LNPs vs. adsorbed to protein.

### 4.7 Cell Culture

Raw 264.7 mouse macrophages were purchased from ATCC (TIB‐71, purchased: 05/14/2024, RRID: CVCL_0493, uncontaminated) and cultured in Dulbecco's modified Eagle's medium (DMEM) with 10% heat‐inactivated fetal bovine serum (FBS) and 1% penicillin/streptomycin (PS).

For 24 well‐plate experiments, RAW 264.7 macrophages were seeded at 400k density using 500 µL volume and treated with 300 µL of LNP volume (diluted in DMEM). For 96 well‐plate experiments, RAW 264.7 macrophages were seeded at 100k density using 100 µL volume and treated with 100 µL of LNP volume (diluted in DMEM). LNP doses for each in vitro experiment can be found in figure legends.

NF‐κB Luciferase Reporter Raw 264.7 Cell Line was purchased from BPS Bioscience INC (79978, purchased: 6/17/2024, uncontaminated) and cultured in Dulbecco's modified Eagle's medium (DMEM) with 10% heat‐inactivated fetal bovine serum (FBS), 700ug/mL Geneticin, 1% GlutaMax, and 1% penicillin/streptomycin (PS). For 96 well‐plate experiments, RAW 264.7 macrophages were seeded at 100k density using 100 µL volume and treated with 100 µL of LNP volume (diluted in DMEM). LNP dosing was done by mRNA amount, and specific doses can be found in figure legends.

THP‐1 Cell Line (RRID: CVCL_0006, uncontaminated) were cultured in RPMI with 10% FBS, 0.05 mm β‐Mercaptoethanol, and 1% penicillin/streptomycin. Cells were differentiated overnight at 0.2 µm of PMA before adding to 96‐well plate and treated as previously described for other cell lines.

HepG2 cell line (RRID:CVCL_0027, uncontaminated) were cultured in DMEM with 10% FBS and 1% penicillin/streptomycin. For 96‐well experiments, cells were seeded at 50k/ well in 100 µL of media. They were left overnight prior to LNP treatment.

### 4.8 In Vitro Luciferase Delivery for Measurements of mRNA Transfection Efficiency

Luciferase mRNA LNPs fabricated as described above were incubated with cells for 6 h. Supernatants were collected (spun down at 10,000 x g for 15 mins) for cytokine analysis using LegendPlex 13‐plex Mouse Inflammation Panel (Biolegend). Adhered cells were lysed with 20 µL of 1X Promega Luciferase Assay Cell Lysis Buffer for 10–15 min on plate shaker ∼300 rpm. The luminescence was read on a luminometer (Promega) after 100 µL of luciferin solution (Promega) was added well by well by an autosampler. Cell viability was measured via Abcam cell counting kit 8 and measured via plate reader on a clear 96 well plate at 450 and 660 nm. Cell viability was normalized to control cells.

### 4.9 In Vivo Delivery for Measurement of Transfection Efficiency and Cytokine Production

Luciferase mRNA LNPs fabricated as described above were injected into mice intravenously or instilled intratracheally for a circulation time of 4 or 2 h, respectively. For intravenous administration, blood was collected using EDTA syringes and tubes and spun down for plasma collection. For intratracheal delivery, bronchoalveolar lavage (BAL) was performed by inserting a catheter intratracheally, dispensing 800 µL of ice cold 1x PBS (0.5 µm EDTA) using 1 mL BD syringe and performing a total of three washes prior to collection. Select organs were then flash‐frozen until the day of analysis or homogenized immediately. Alternatively, luciferase mRNA LNPs were injected into shaved mice intradermally for 24 h. ∼1cm [2] of skin was collected, weighed and then flash‐frozen until the day of analysis or homogenized immediately.

Organ samples were suspended in 900 µL of homogenization buffer (5 mm EDTA, 10 mm EDTA, 1:100 diluted stock protease inhibitor (Sigma), and 1x (PBS), samples were then loaded with a steel bead (Qiagen), then placed in a tissue homogenizer (Powerlyzer 24, Qiagen) using the following settings: Speed (S) 2000 rpm, 2 Cycles (C), T time 45 sec, and pause for 30 sec). After this, 100 µL of lysis buffer (10% Triton‐X 100 and PBS) was added into each tube and then allowed to incubate for 1 h at 4C. After this, they were immediately transferred into fresh tubes, and sonicated, using a point sonicator to remove excess DNA, using an amplitude of 30%, 5 cycles of 3 s on/off. After this, samples were then centrifuged at 16000 x g for 10 min. The resultant lysate was either frozen or prepared for luminometry analysis or cytokine measurement.

For luciferase expression, 20 µL of undiluted sample was loaded onto a white 96 well‐plate and the luminescence was read on a luminometer (Promega) after 100 µL of luciferin solution (Promega) was added well by well by an autosampler.

For cytokine analysis, organ homogenates, BAL, and plasma were collected and measured using LegendPlex 13‐plex Mouse Inflammation Panel (Biolegend). The data for this manuscript were generated in the Penn Cytomics and Cell Sorting Shared Resource Laboratory at the University of Pennsylvania (RRID:SCR_022376). Penn Cytomics is partially supported by the Abramson Cancer Center NCI Grant (P30 016520).

### 4.10 In Vivo Imaging System (IVIS) for Luciferase Expression

Prior to IVIS imaging, mice were intravenously injected with LNPs ± small molecule inhibitors and were loaded with mRNA encoding for Luciferase (Aldevron) at a dose of 10 µg. 4 h post treatment, mice were put under using 3% isoflurane‐induced and intraperitoneally injected with 100 µL of 30 mg/mL D‐luciferin sodium salt (Regis Technologies). Anesthetized mice were placed in an IVIS Spectrum machine (Revvity) face up and imaged for chemiluminescence in the target area every minute with automatically determined exposure time for 10–14 images, until the signal reached the peak intensity. Revvity LivingImage software (version 4.8.2) was used to analyze images.

### 4.11 Ex Vivo Imaging

Following IVIS imaging 4 h post LNP administration, mice that were already intraperitoneally injected with 100 µL of 30 mg/mL D‐ luciferin sodium salt (Regis Technologies) were anesthetized and euthanized. Organs (lungs, liver, spleen, heart, and kidney) were collected, organized on a 10 cm dish, and imaged for chemiluminescence in an IVIS Spectrum machine (Revvity). Revvity LivingImage software (version 4.8.2) was used to analyze images.

### 4.12 Liver/Spleen Flow Cytometry

Naive mice were intravenously injected with mRNA‐LNPs formulated with 1% 18:1 PE‐TopFluor AF594 (Avanti Polar Lipids), to track particle delivery. Either 30 min or 24 h (mCherry LNPs) after treatment, mice were euthanized and transcardially perfused with PBS to clear blood and preserve tissue. The liver and spleen of the mice were collected and triturated before being incubated in a digestive solution of 2 mg/mL type 1 collagenase (Gibco) and 100 µL of 2.5 mg/mL DNAse (Roche) for 45 min to prepare a single‐cell suspension. After incubation, the cells were strained through a 70 µm cell strainer (Sycamore Life Science) and washed with PBS. The supernatant was discarded, and ACK lysis buffer (Gibco) was added for 5 min on ice to lyse any remaining red blood cells (RBCs). After RBC lysis, an automated cell counter (Countess, Thermo Fisher) was used to achieve a final cell concentration of 1 ×10^6^ cells/mL for each sample. Suspensions were washed with Fluorescence‐activated Cell Sorting (FACS) buffer, consisting of PBS, 1% Fetal Bovine Serum (FBS, Thermo Fisher Scientific), and 1 mm Ethylenediaminetetraacetic Acid (EDTA, Invitrogen), and incubated with anti‐CD16/CD32 monoclonal antibodies (Invitro‐ gen) for 15 min to prevent nonspecific binding of antibodies on Fc‐receptors. Following washing, suspensions were incubated for another 30 mins with an antibody cocktail consisting of brilliant ultraviolet (BUV) 395 anti‐mouse CD45, clone: 30‐F11 (BD Bio‐ sciences), phycoerythrin (PE)/cyanine7 anti‐mouse‐CD64, clone: Fc *γ*RI (BioLegend), and Alexa Fluor 700 anti‐mouse Ly6G, clone: Gr‐1 (BioLegend) to identify immune cell subpopulations, and Fluorescein isothiocyanate (FITC) anti‐mouse CD86, clone: A17199A (Biolegend) to identify immune cell subpopulations, and allophycocyanin (APC) anti‐mouse CD31 (BioLegend) and brilliant violet 711 (BV711) anti‐mouse Ep‐CAM, clone: CD326 (BioLegend) for endothelial and epithelial cells. After antibody incubation, the cells were fixed with 4% paraformaldehyde (PFA, Electron Microscopy Sciences) before analysis on a flow cytometer (LSR Fortessa, BD BioSciences). Gating parameters for analysis are illustrated in Figure S16. The flow cytometry results were analyzed using FlowJo Software v10.10 (BD Life Sciences).

### 4.13 Statistics

All results are expressed as mean ± SEM unless specified otherwise. Statistical analyses were performed using GraphPad Prism 8 (GraphPad Software) * denotes *p*<0.05, ^**^ denotes *p*<0.01, ^***^ denotes *p*<0.001, ^****^ denotes *p*<0.0001.

## Funding

Grants used to fund this work include NIH grants R01‐HL‐153510, R01‐HL160694, R01‐HL164594, and R41‐NS‐130812.

## Conflicts of Interest

We have submitted a provisional patent on loading NF‐κB inhibitors into LNPs. There are no other conflicts of interest to report.

## Supporting information




**Supporting File**: advs74929‐sup‐0001‐SuppMat.pdf.

## Data Availability

The data that support the findings of this study are available from the corresponding author upon reasonable request.
