# Supplementary material for: Localized NF‐κB Inhibition Reduces Lipid Nanoparticle‐Associated Inflammation
Source: Adv Sci (Weinh). 2026 Mar 24;13(31):e17931. doi: 10.1002/advs.202517931 (PMC13587984; doi:10.1002/advs.202517931)

Localized NF-κB Inhibition Reduces Lipid Nanoparticle-Associated Inflammation

Carolann L Espy[1], Jichuan Wu[2], Serena Omo-Lamai[3], Liuqian Wang[3], Fengyi Dong[3], Aleksa Milosavljevic[2], Sarah O'Neill[1], Taylor Brysgel[1], Yufei Wang[1], Michael Zaleski[1], Emily Wolfe[4], Rhea Maheshwari[2], Manthan Patel[1], Dirk Trauner[1], Jia Nong[1], Zhicheng Wang[1,2]*, Jacob S Brenner[1,2]**

* To whom correspondence should be addressed

Supporting Information

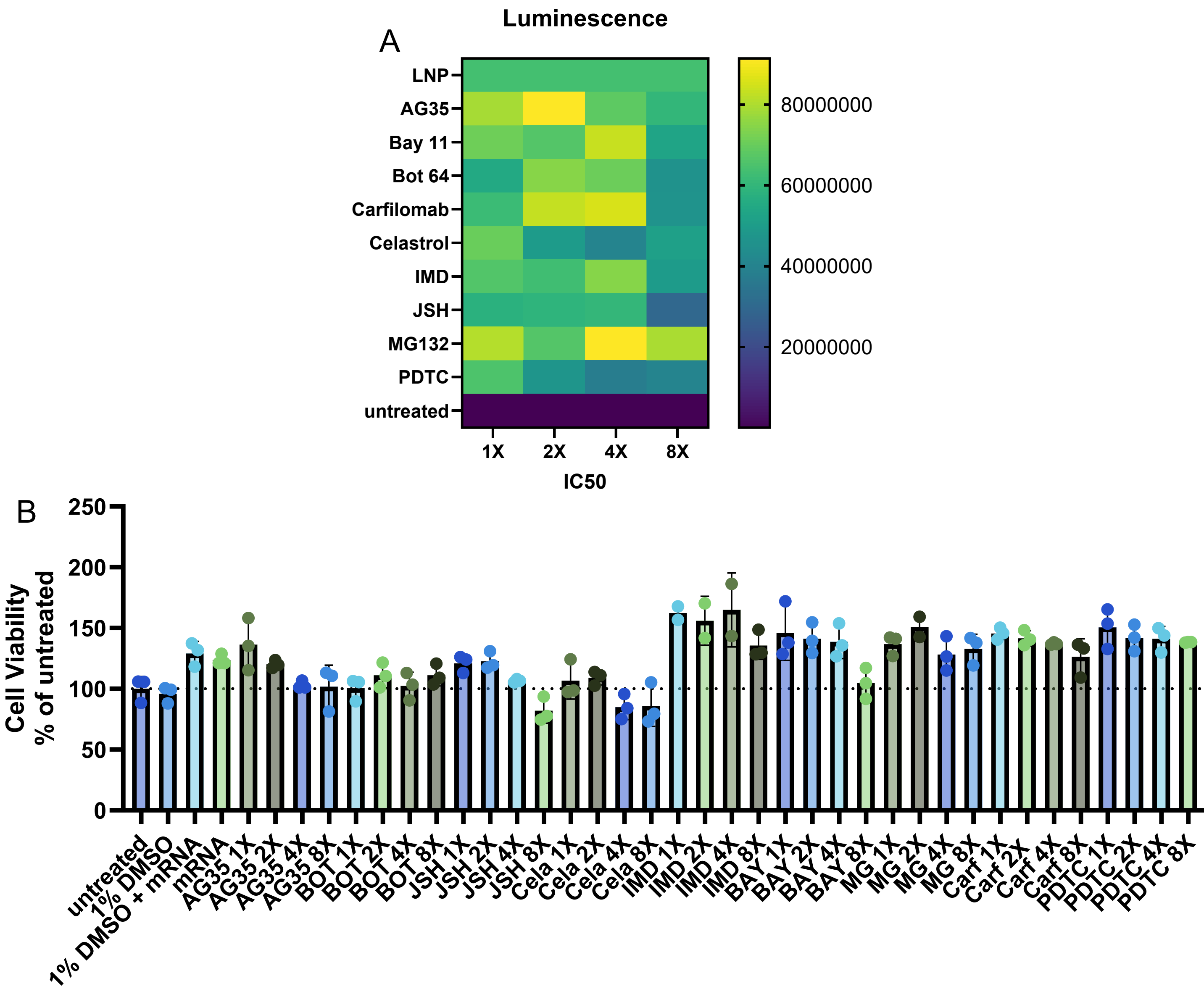

Supplementary Figure 1. Free small molecule inhibitors plus LNP treatment on RAW macrophages show relatively no difference in luciferase mRNA expression (A) and cell viability (B). Luminescence and cell viability were measured 6 hours after initial LNP treatment. Luminescence was measured via luminometer, and cell viability was measured using cell counting kit 8. Statistics: For A and B n=3 biological replicates. A data shown represents mean and B represents mean ± SEM.

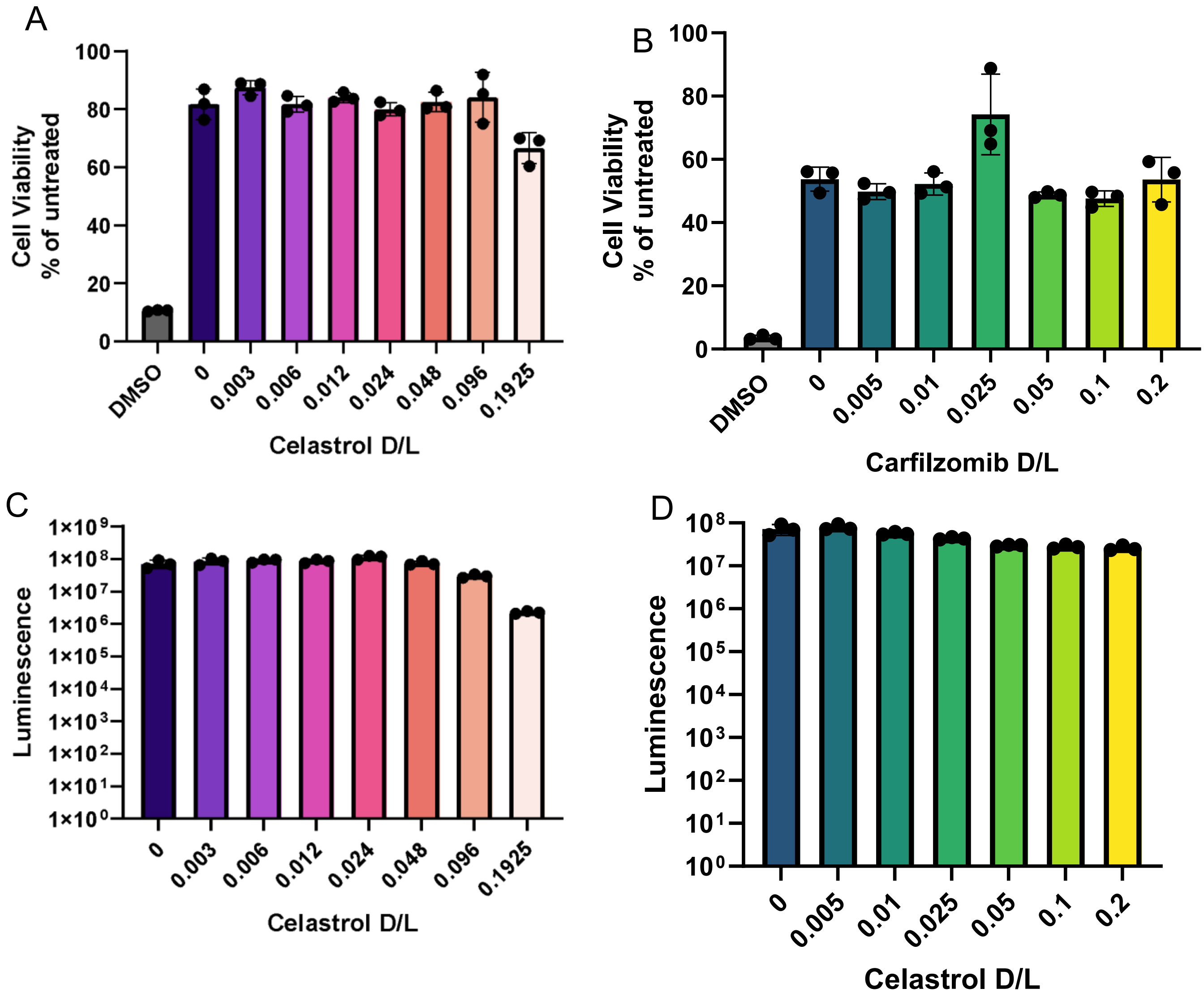

Supplementary Figure 2. Celastrol- and carfilzomib-LNPs do not significantly decrease cell viability or expression except at celastrol's highest dose. RAW Macrophages were treated for six hours with 98N12 Fluc mRNA LNPs containing either celastrol or carfilzomib at a dose of 1000 ng/mL mRNA. (A) and (B) show similar cell viability levels of cells treated with just LNPs (0 D/L) and LNPs with celastrol and carfilzomib. (A) Only the highest dose of celastrol showed a decrease in cell viability compared to LNP only treated group. (C) and (D) show that LNPs containing celastrol and carfilzomib are still able to express their nucleic acid cargo. Statistics: For A-D, n = 3 and data shown represents mean ± SEM.

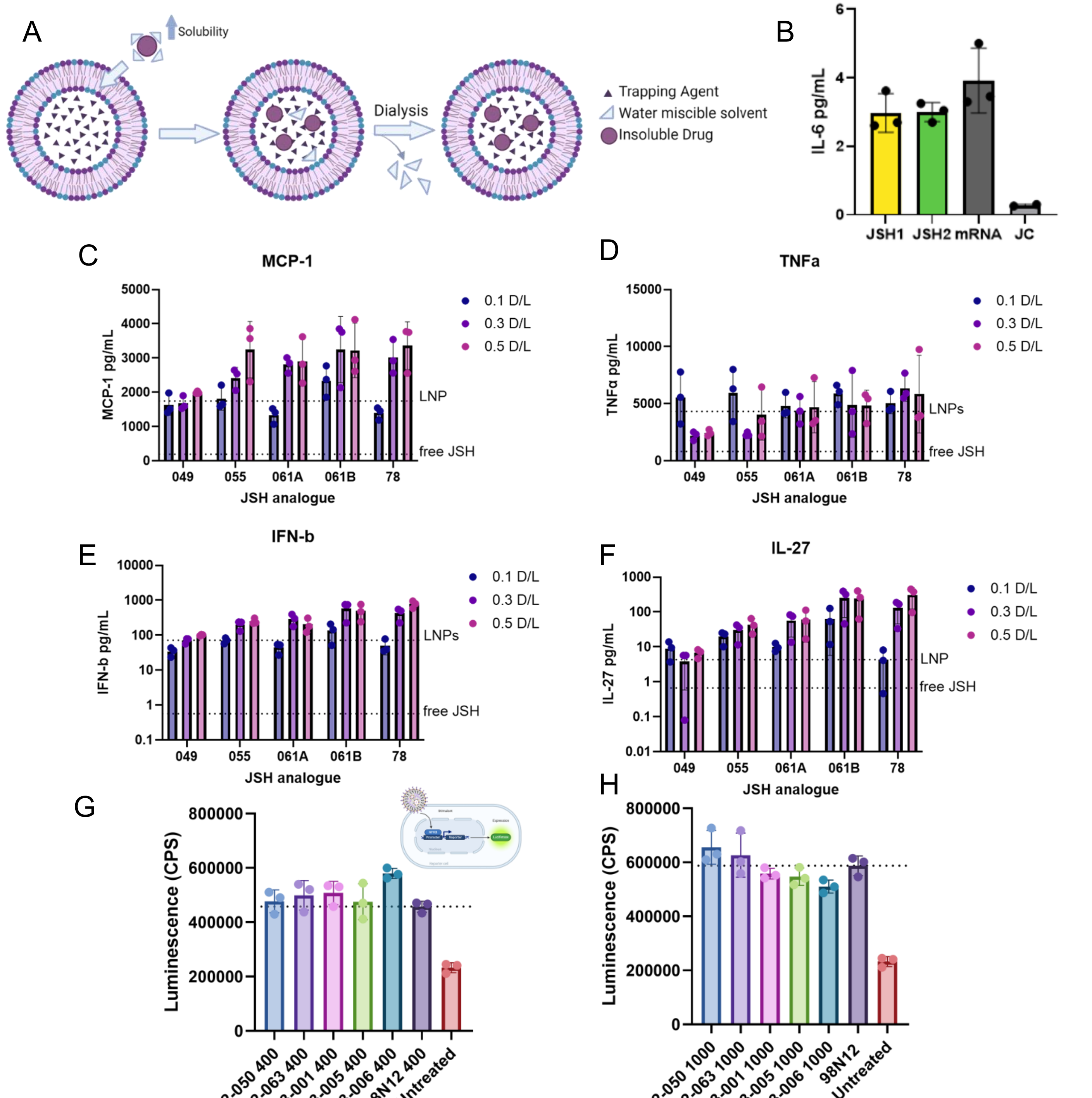

Supplementary Figure 3. Active loading and pro-drug forms of JSH do not load into LNPs and are unable to resolve LAI. (A) Active loading strategy was applied to load JSH-23 into liposomes. After purification of free drug, no drug was measured in LNPs via UPLC. (B) This is supported by the lack of efficacy in reducing inflammatory cytokines with the active loaded liposomes in vitro. RAW Macrophages treated for six hours at a dose of 1000 ng/mL of mRNA. (C-F) Show a pro-drug approach where JSH-23 was synthesized with a lipidic tail to assist with LNP loading. Cytokine read outs indicate a lack of efficacy of the lipidic-JSH. (G-H) Show lipid pro-drug of JSH loaded into 98N12 LNPs with mCherry and delivered to NFKB RAW Macrophage reporter line with increased luminescence indicating increased NFKB activity. At a dose of 400ng/mL mRNA (G) and 1000 ng/mL mRNA (H) there was very little difference in NFKB activity as measured by luminescence. Statistics: For (B-H), n=3 and data shown represents mean ± SEM.

Supplementary Figure 4. pDNA LNPs ± celastrol show significant reduction in expression of firefly luciferase pDNA as celastrol increases. For A, n = 3 and data shown represents mean ± SEM. Comparisons were made using one-way ANOVA with Tukey's post-hoc test. **** = p<0.0001

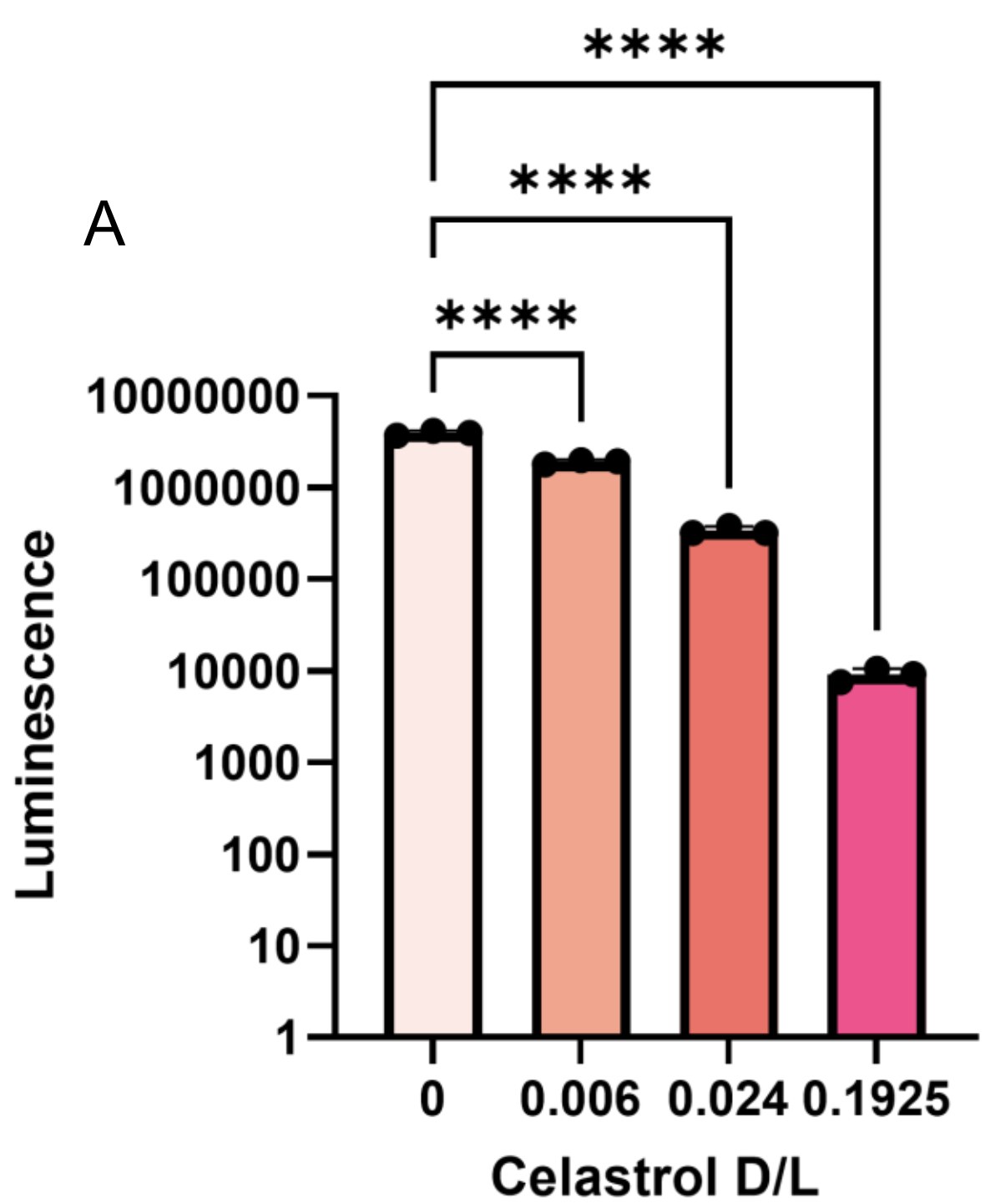

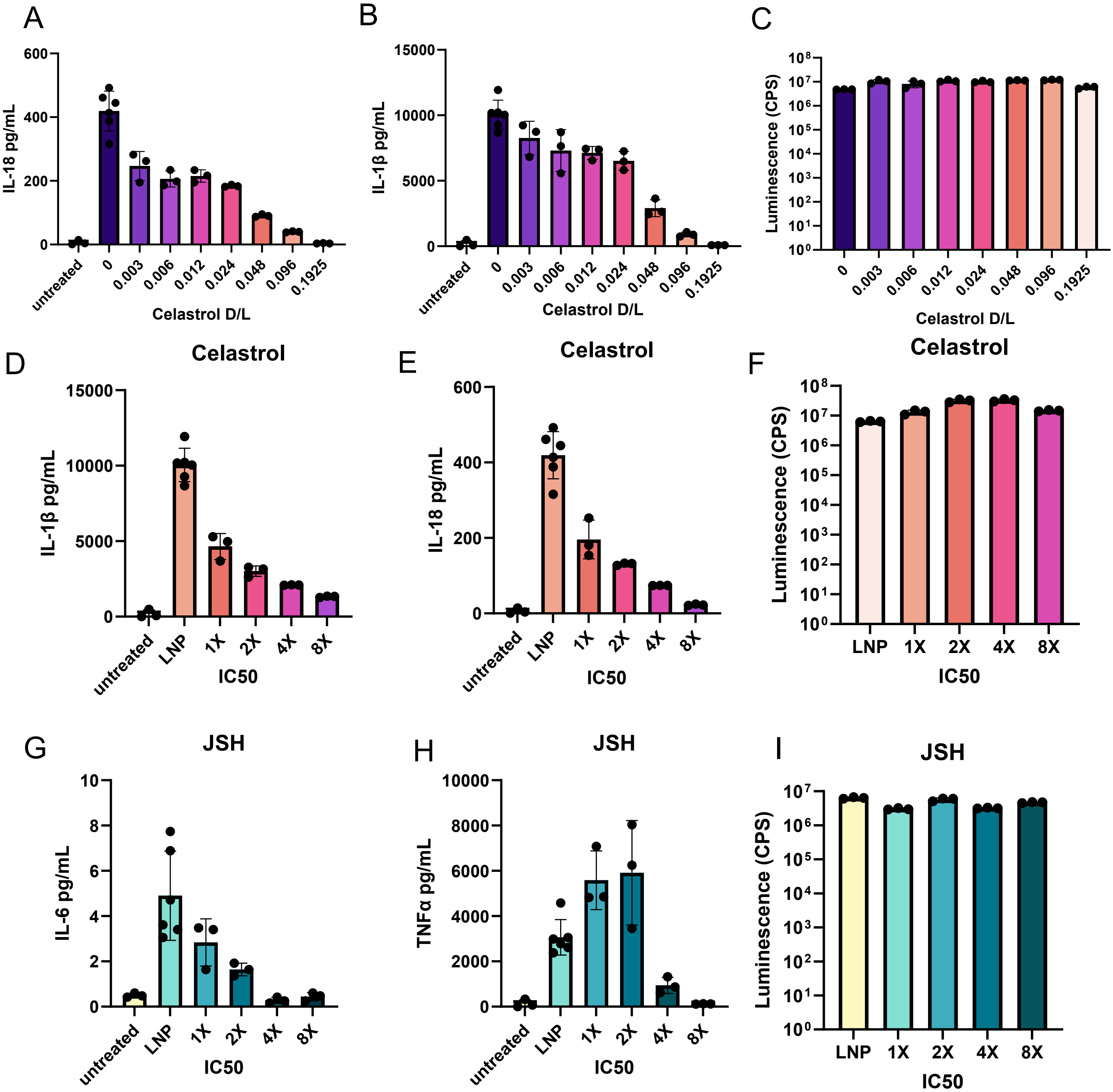

Supplementary Figure 5. Celastrol and JSH anti-inflammatory effects are maintained in differentiated human THP-1 cells. THP-1 cells were treated for with 98N12 LNPs at a dose of 1000ng/mL mRNA for 6 hours. Cytokines IL-1β and IL-18 show clear dose dependent decrease for celastrol when it is encapsulated in the LNP (A-B) or cotreated as a free drug (D-E). Similarly, JSH shows a dose dependent inhibition of cytokine secretion of IL-6 and TNFα (G-H). Neither celastrol nor JSH disrupted expression of mRNA cargo (C, F, I). Statistics: For A-I, n = 3 and data shown represents mean ± SEM.

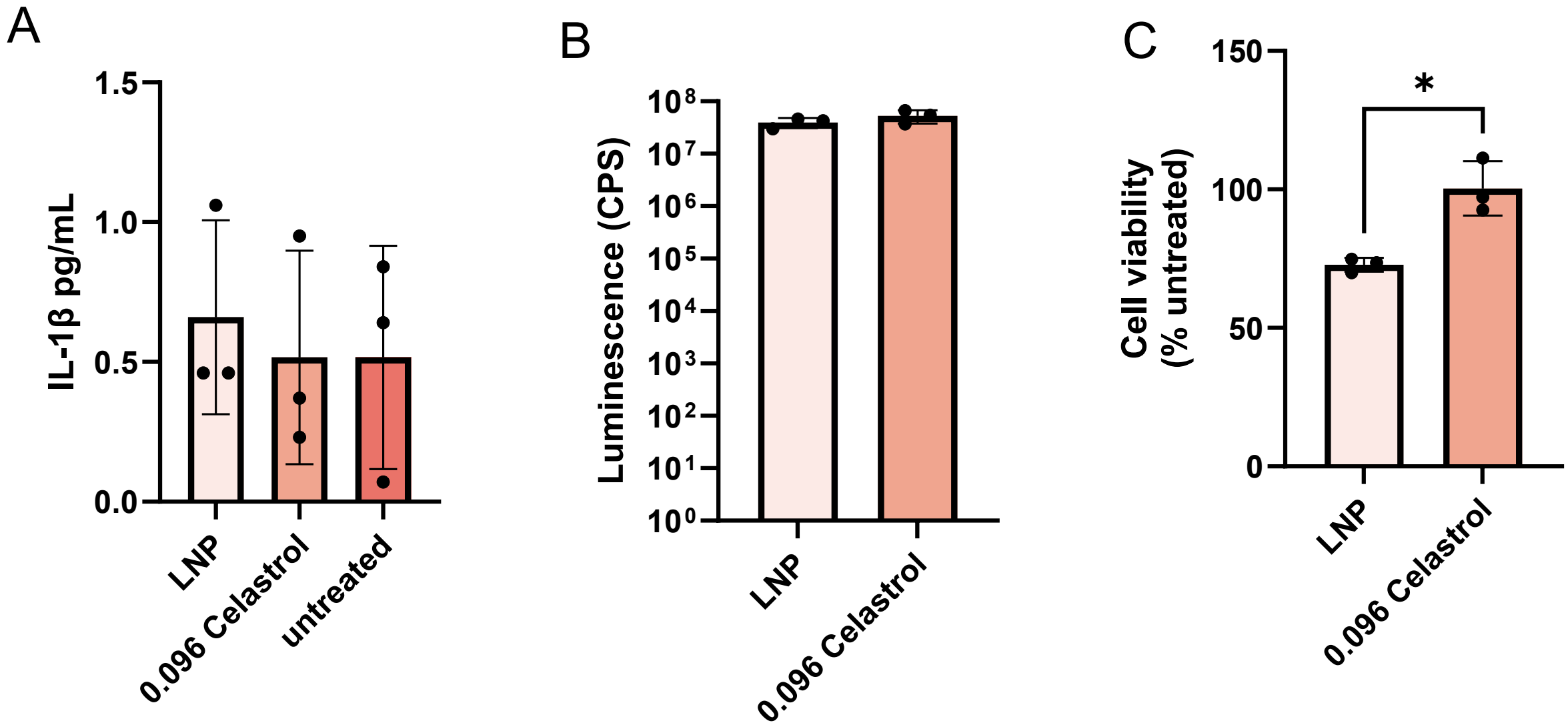

Supplementary Figure 6. Hep G2 cells do not secrete cytokines when treated with 98N12 LNPs. Hep G2 cells were treated with 98N12 LNPs at a dose of 1000ng/mL for 6 hours. (A) Despite the same dose and similar mRNA expression levels as seen in THP-1 cells, Hep G2 cells do not secrete cytokines to a detectable level. (B) Addition of celastrol in the LNP formulation does not decrease mRNA expression. (C) There may be a slight protective effect against cytotoxicity when celastrol is included in the LNP formulation. Celastrol LNPs with a D/L of 0.096 had an average cell viability of 100%, while the cell viability of LNPs without celastrol was 77%. Statistics: For A-C, n = 3 and data shown represents mean ± SEM. (C) comparisons were made using an unpaired t-test with Welch's correction. * = $p<0.05$

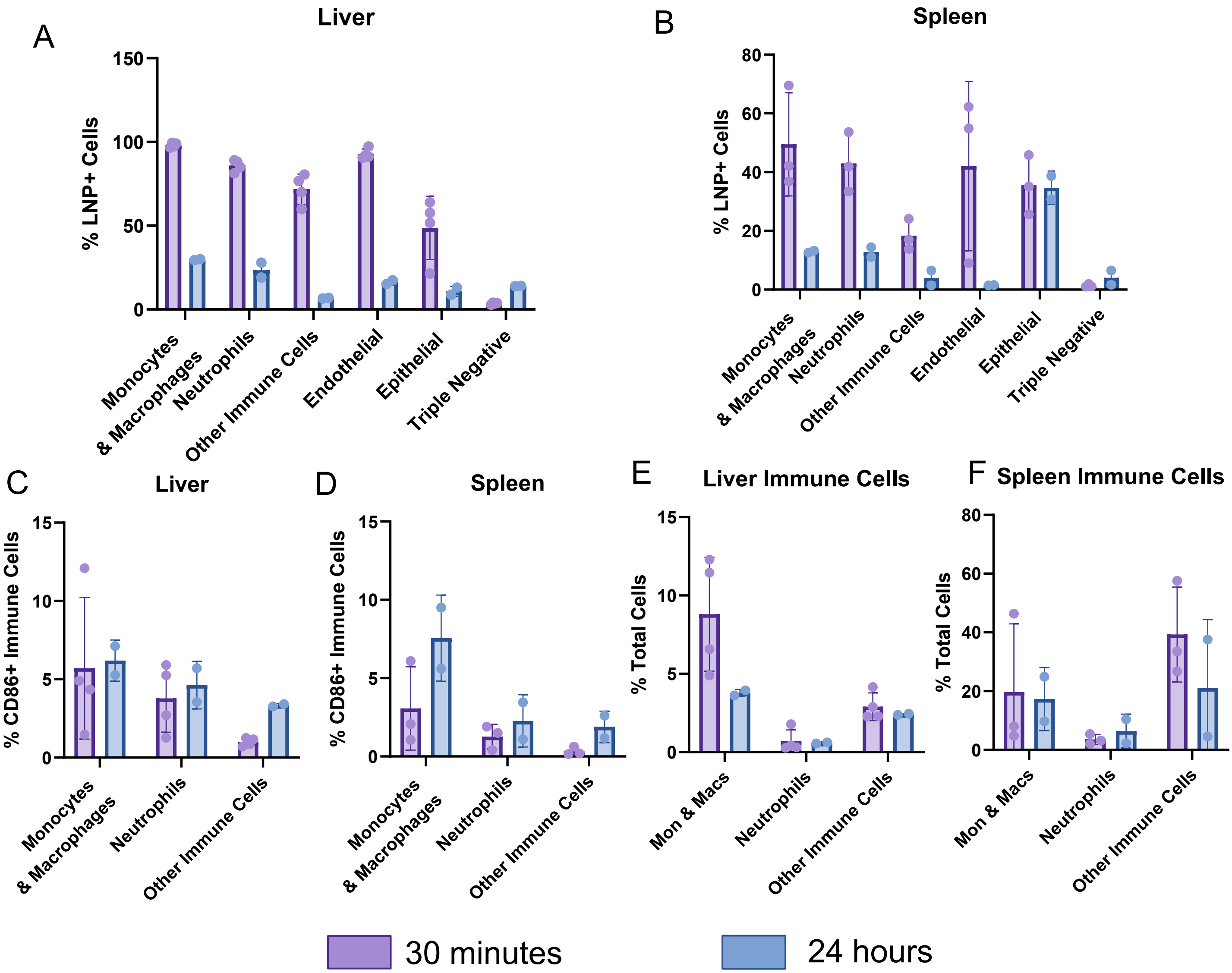

Supplemental Figure 7. Flow cytometry shows high levels of uptake by immune cell populations in the liver and spleen. Fluorescent 2-stage mixing LNPs were administered at a dose of 0.4 mg/kg (~10ug) per mouse. Mice were sacrificed at 30 minutes or 24 hours and processed for flow cytometry. (A,B) Both liver and spleen saw high levels of LNP uptake in immune cell populations. (C-D) there was little difference in CD86 positivity of immune cells between 30 minutes and 24 hours. (E-F) Additionally, there is little change in the percentage of immune cells in the total population of cells collected. Statistics: For A-F, n = 2-4 and data shown represents mean ± SEM.

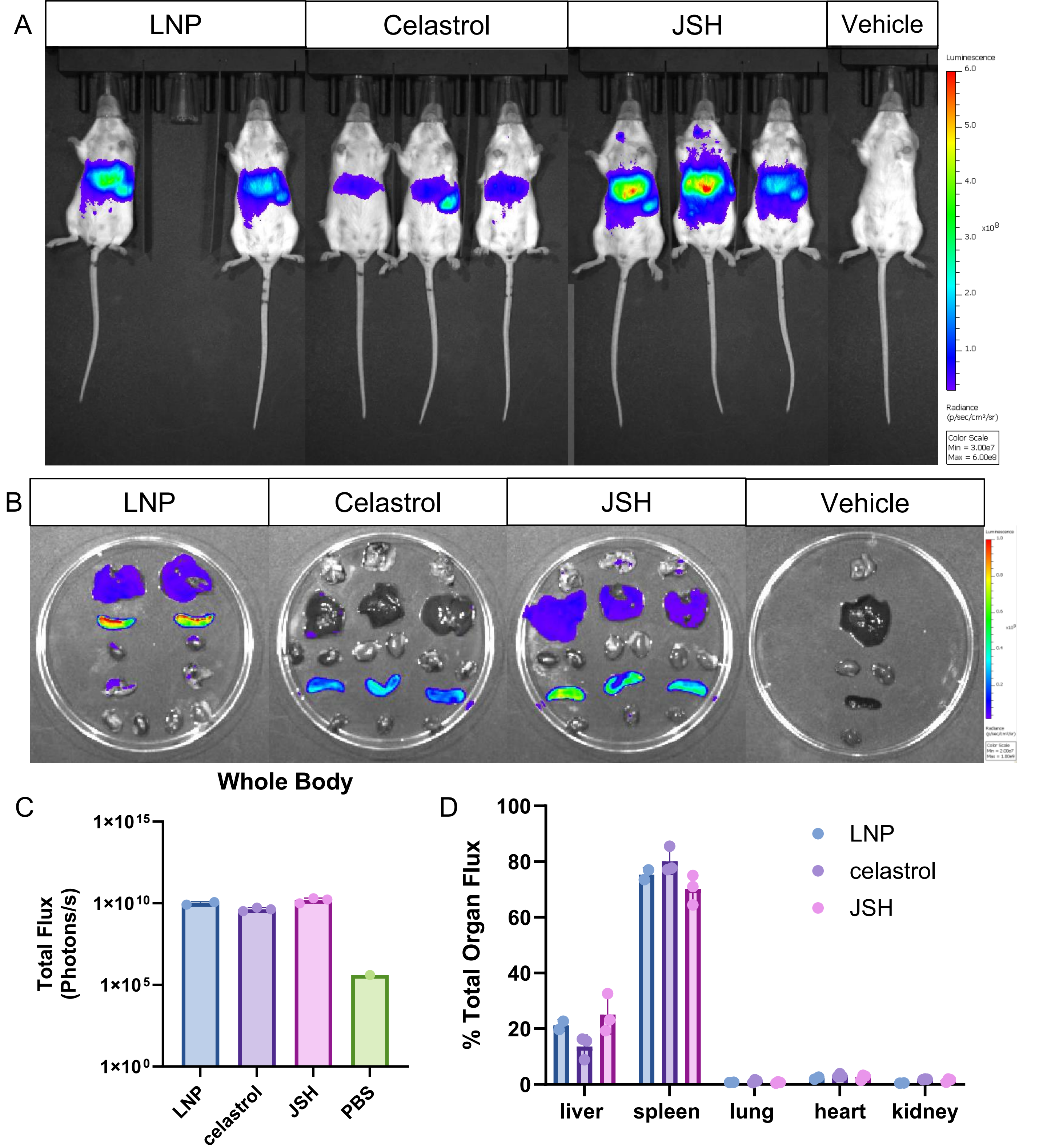

Supplementary Figure 8. Addition of small molecule inhibitors has minimal change on LNP distribution. Mice we administered with 10ug (0.4mg/kg) of mFluc LNPs for four hours, injected with luciferin and imaged via IVIS. (A) Shows whole body images of mice after treatment of LNPs with or without small molecule inhibitors encapsulated. (B) Images of ex vivo organs show majority of signal in the spleen and liver. Organ order: lung, liver, kidneys, spleen, heart for Celastrol, JSH, and Vehicle conditions; liver, spleen, heart, lung, and kidney for LNP condition. (C) Total body ROI indicates little difference in luciferase mRNA expression between the different LNPs. (D) ROI by organ shows similar distribution between the different formulations again with majority of the signal being in spleen followed by liver. Statistics: For A-D, n = 1-3 and data shown represents mean ± SEM

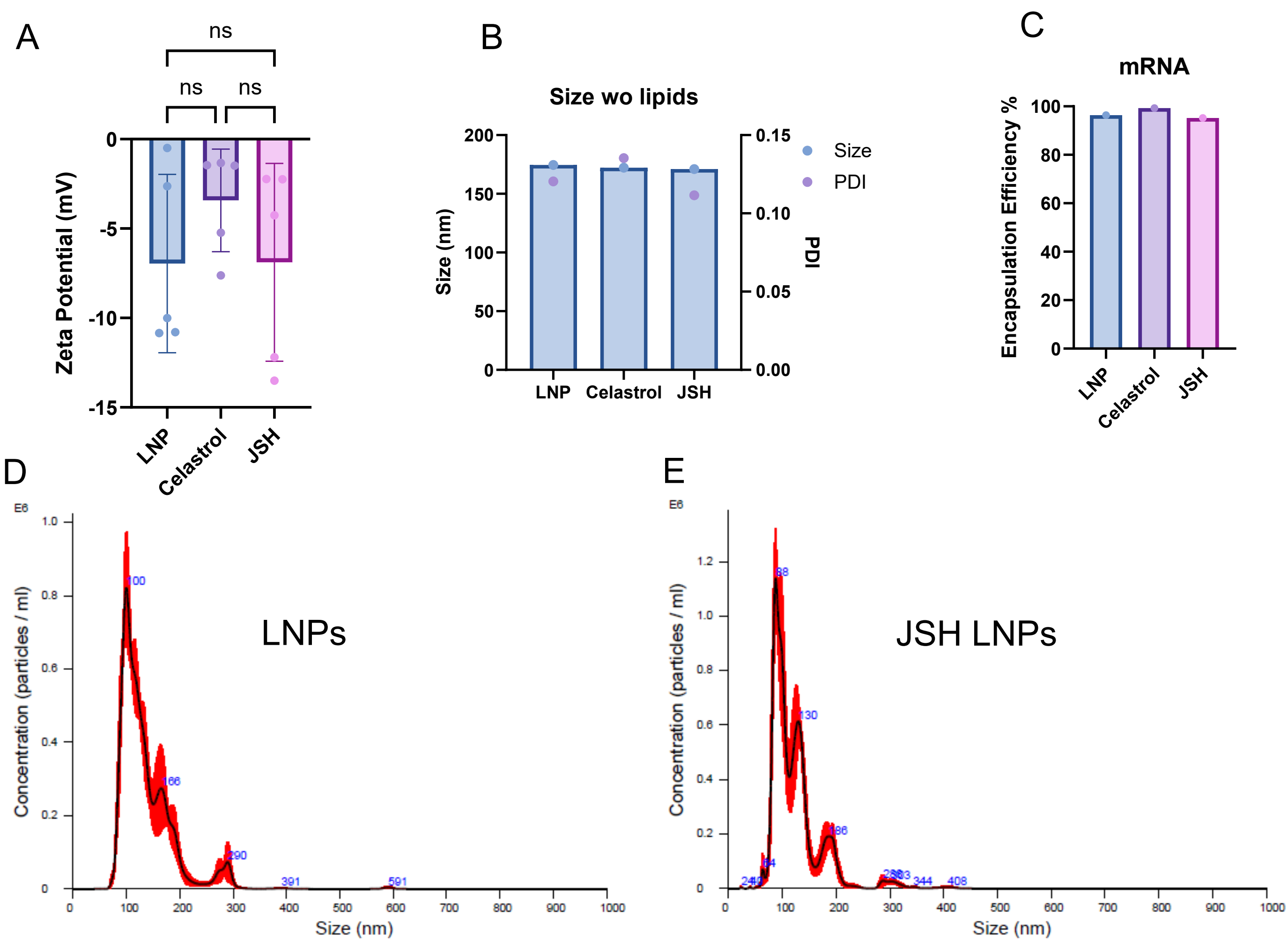

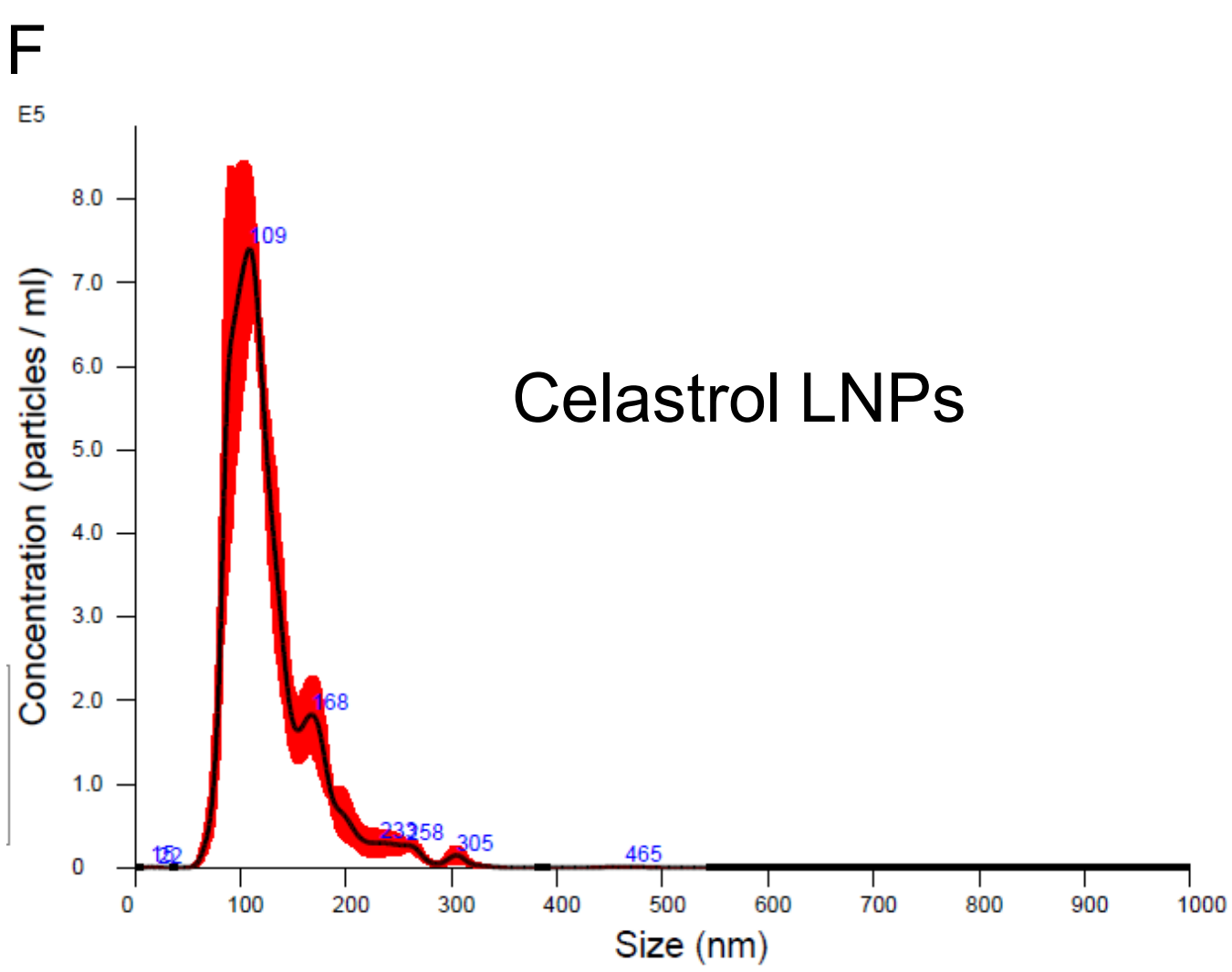

Supplementary Figure 9. 2-step mixing LNP characterization. (A-B) No significant difference in zeta potential or LNP size as measured via DLS. (C) mRNA encapsulation was unchanged regardless of small molecule inclusion in the formulation. (D-F) Concentration (particles/mL) versus particle size (nm) show little difference between the LNP formulations as measured via nanoparticle tracking analysis (NTA). Statistics: For A, D-F, n = 5 and data shown represents mean ± SEM. (A) Comparisons were made using one-way ANOVA with Tukey’s post-hoc test.

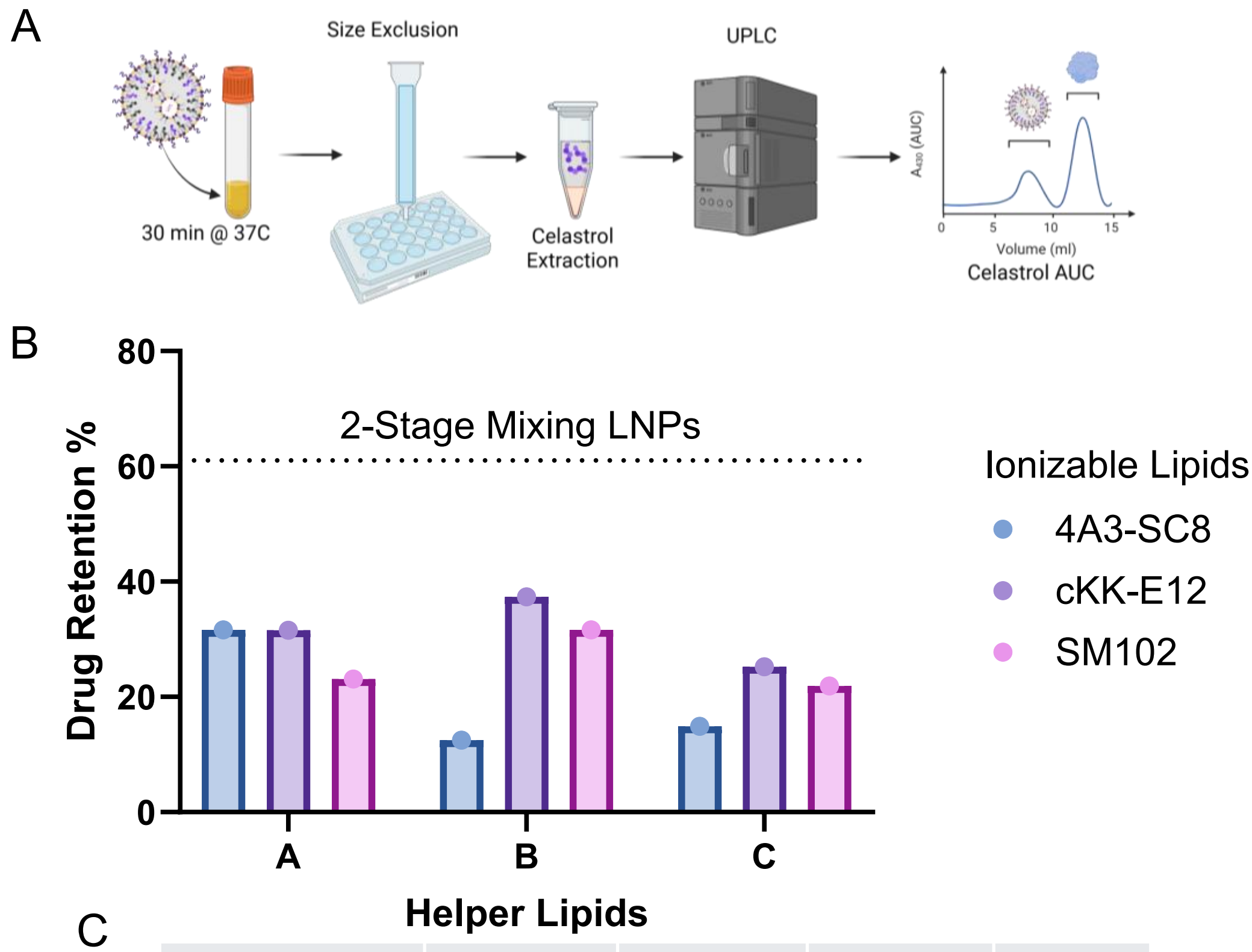

C

	A	B	C	2-Stage Mixing
Ionizable Lipid	50%	50%	50%	50%
Cholesterol	38.50%	38.50%	38.50%	38.50%
DOPE	10%	10%		
DSPC			10%	10%
DMG-PEG2k	1.50%			1.50%
DSPE-PEG2k-Az		1.50%	1.50%	

Supplementary Figure 10. Celastrol Retention post plasma incubation. (A) Method for measuring celastrol leak after plasma incubation. (B) Both helper lipids and ionizable lipids impact drug retention in plasma. Two-step mixing had a greater impact on drug retention compared to changes in the LNP formulation. Two-step mixing resulted in >60% celastrol retention post plasma incubation while changes in lipid composition resulted in a maximum retention of ~37%. (C) Formulations for the LNPs tested in B. Created in BioRender. Brenner, J. (2026) https://BioRender.com/4jf9ur8

A

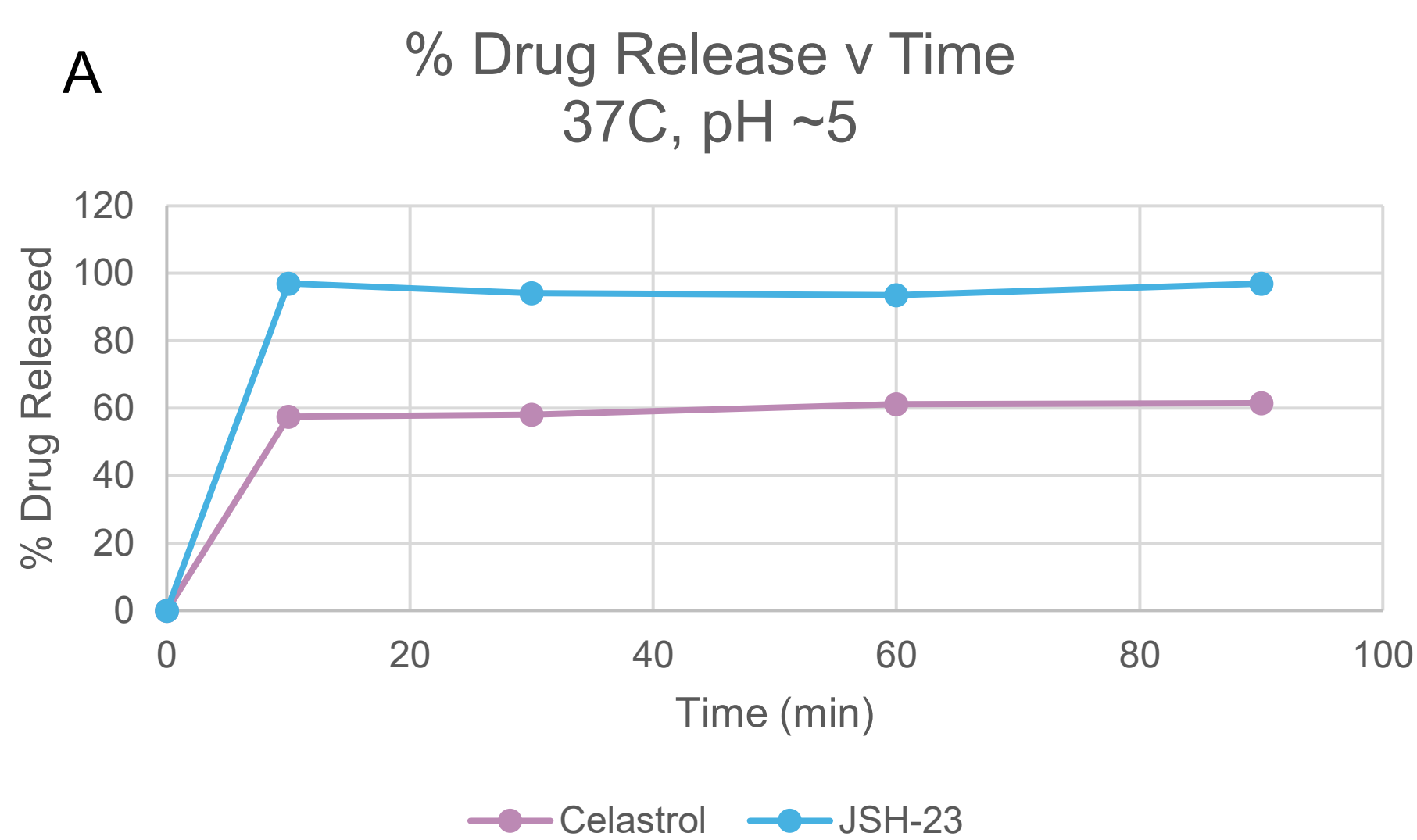

Supplementary Figure 11. Drug release in endosome/ lysosome like conditions. LNPs were incubated in low pH environment at 37C for various lengths of time. LNPs were then run through a zeba column to remove free drug and measured on UPLC for drug concentration. (A) Shows nearly 100% release within 15 minutes for JSH and ~60% release for celastrol.

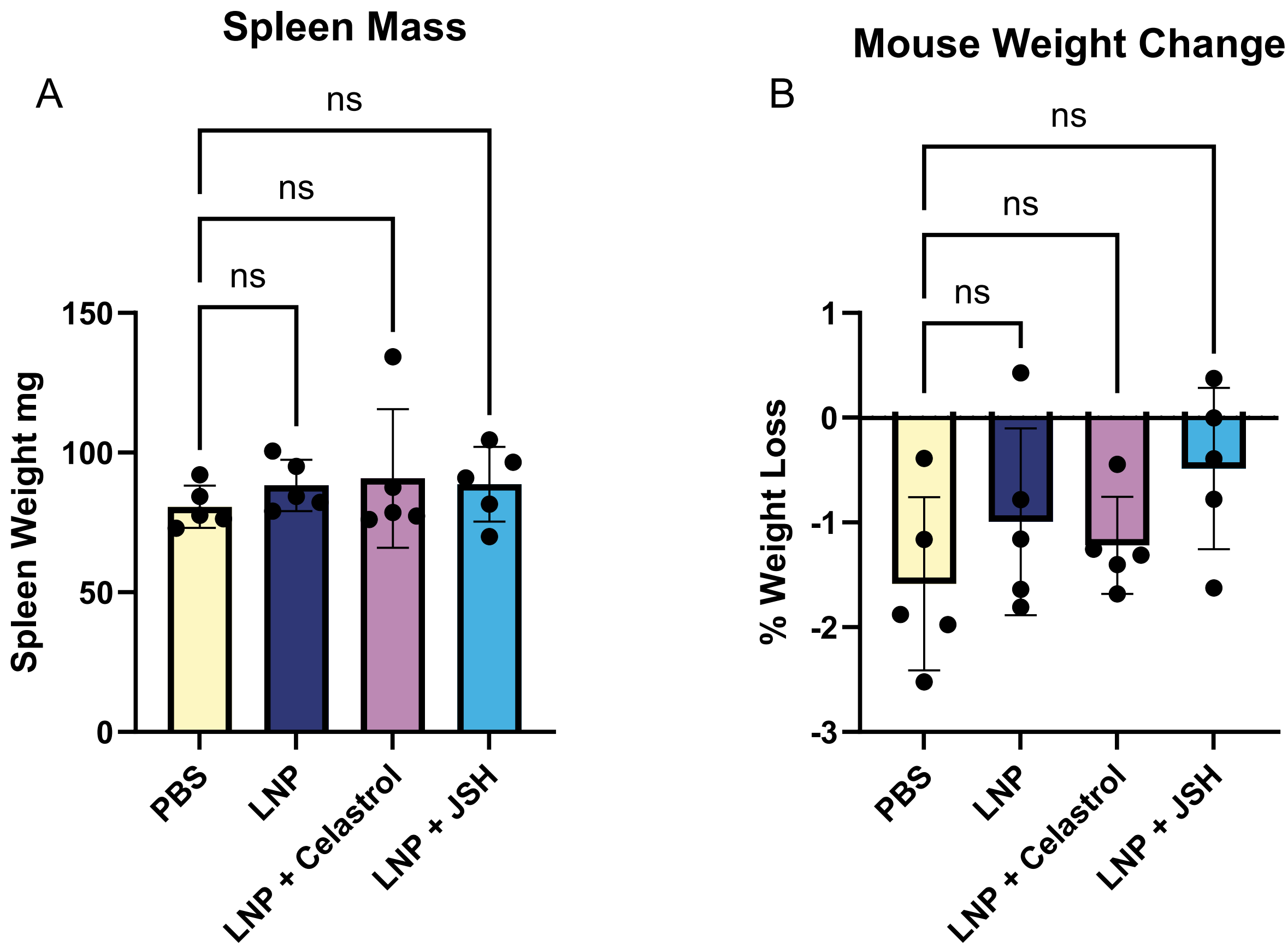

Supplementary Figure 12. No difference seen in spleen weight (A) or mouse weight change (B) 4 hours after LNP administration. Increase in spleen weight and negative weight loss are indications of toxicity in mice. Statistics: For A-B, n = 5 and data shown represents mean ± SEM. Comparisons were made using one-way ANOVA with Tukey's post-hoc test.

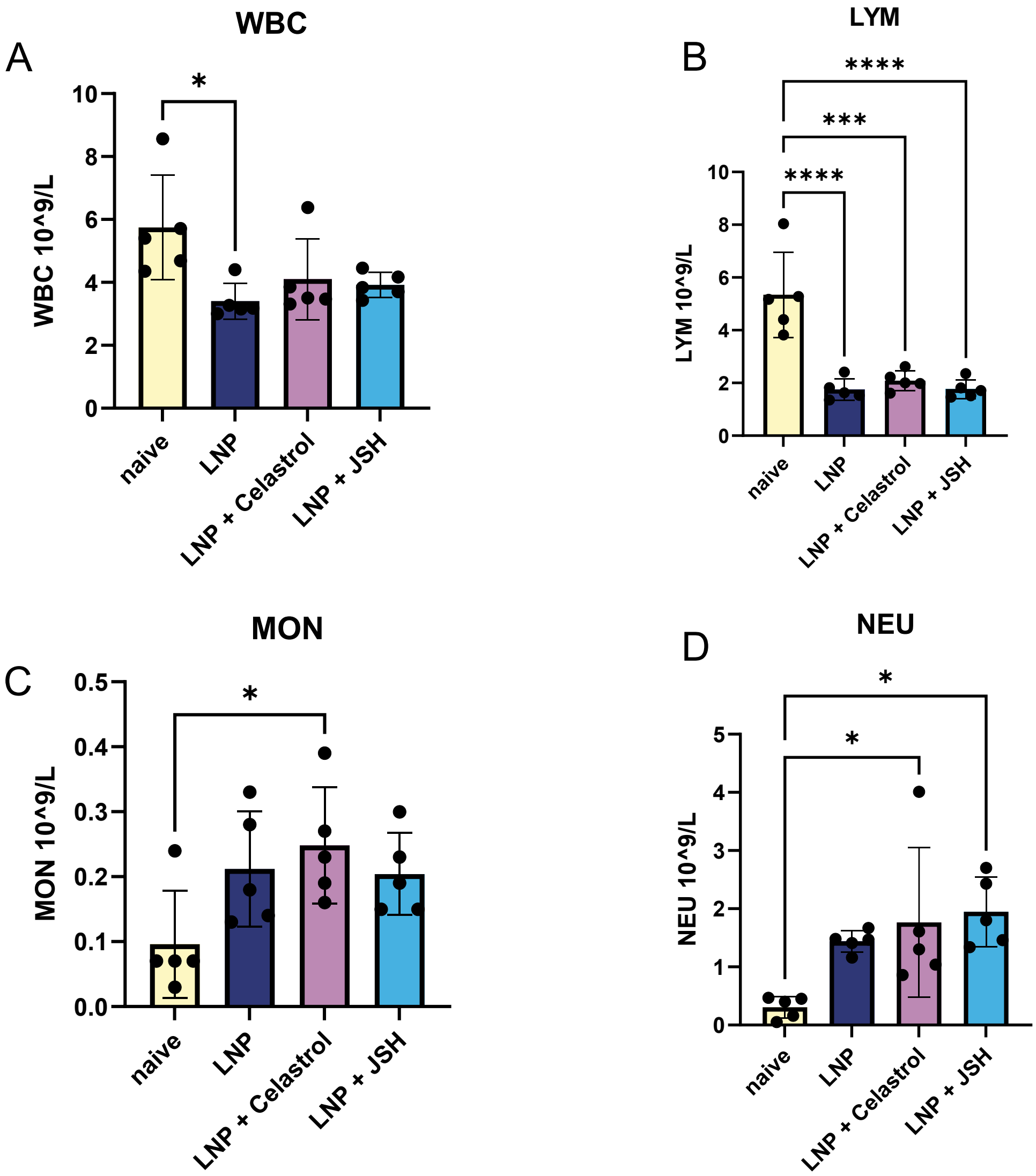

Supplementary Figure 13. Complete blood count four hours after intravenous administration of 10µg LNPs. (A) LNP treated mice show statistically significant decrease in white blood cell count, which is not seen in celastrol and JSH loaded nanoparticles. (B) Severe lymphopenia is caused by LNP administration. (C-D) see an increase in circulating monocytes and neutrophils for celastrol LNPs. Statistics: For A-D, n = 5 and data shown represents mean ± SEM. Comparisons were made using one-way ANOVA with Tukey's post-hoc test.

A

	A	B	C	D
98N12	50%			
SM-102		50%	46%	46%
Cholesterol	38.5%	38.5%	35.3%	34.3%
DOPE	10%		6.5%	6.5%
DOTAP			1.7%	1.7%
DSPC		10%	9.2%	9.2%
DMG-PEG2k	1.5%	1.5%	1.4%	1.4%
AF594				1%
N:P	~6.8	~10	~10	~10

B

Formulation	Main Figures	Supplementary Figures
A standard mixing	Figure 1F-J, Figure 2, Figure 3A-J, Figure 4A-D, Figure 5A-E	Sup Figure 1, 2, 3, 5, 6
B standard mixing	Figure 1A-C, Figure 3K, Figure 4F-K	Sup Figure 4, 10
B two-step mixing	Figure 4G-K, Figure 5F-Y	Sup Figure 9, 10, 11, 12, 13, 15
C two-step mixing		Sup Figure 8
D two-step mixing		Sup Figure 7

Supplementary Figure 14. Table (A) LNP formulations used in main figures 1-5. LNPs were made with a lipid weight to mRNA weight ratio of 40:1. This is equivalent to an ionizable lipid to mRNA molar ratio of ~6.8 for formulation A and ~10 for formulation B. Table (B) Indicates the formulations used in each figure. Formulations C and D are early iterations of the final two-step mixing formulation B.

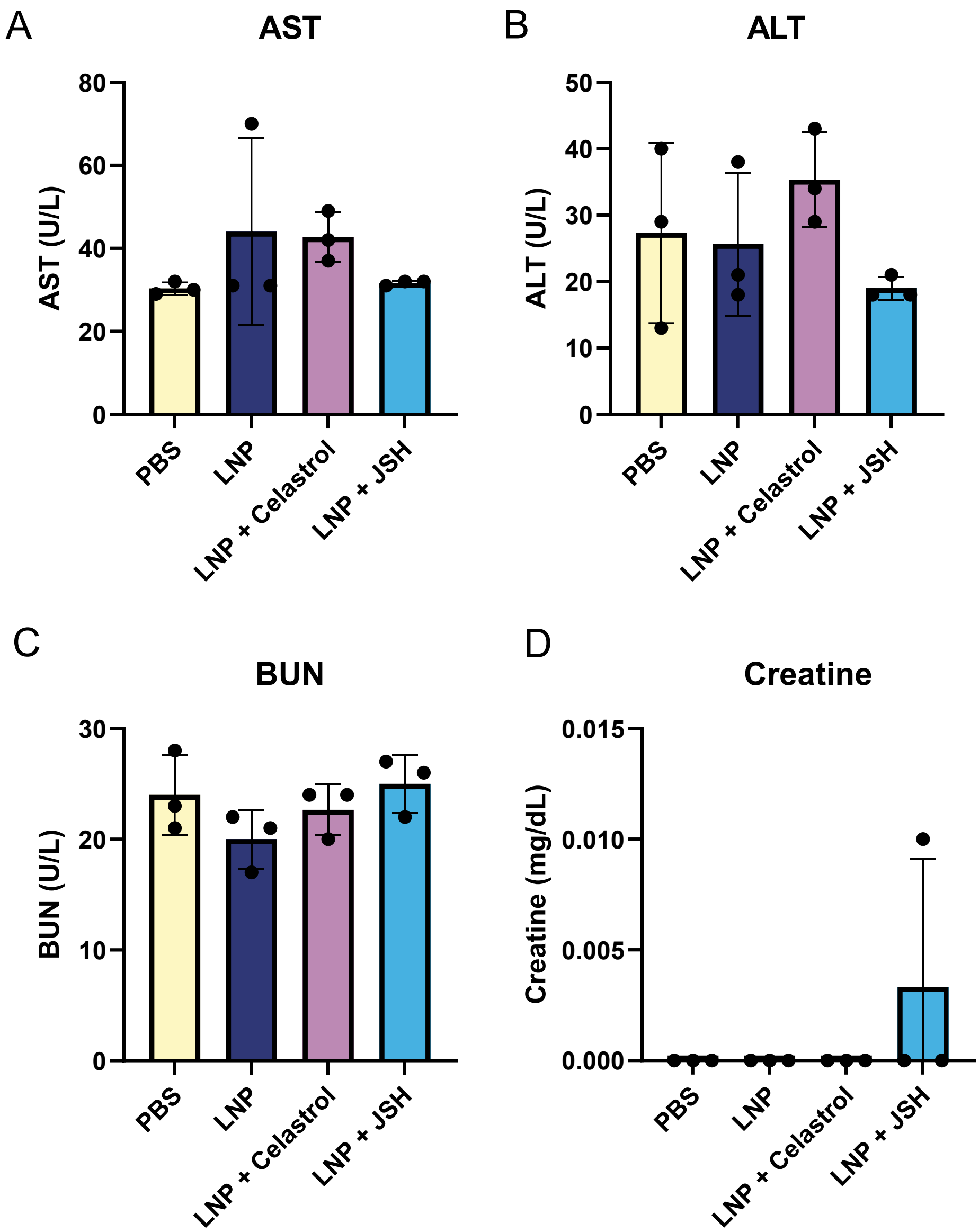

Supplementary Figure 15. No significant elevation of liver (A-B) and kidney (C-D) damage markers in LNP treated mice compared to PBS vehicle 24 hours after IV administration. Statistics: For A-D, n = 5 and data shown represents mean ± SEM. Comparisons were made using one-way ANOVA with Tukey's post-hoc test.

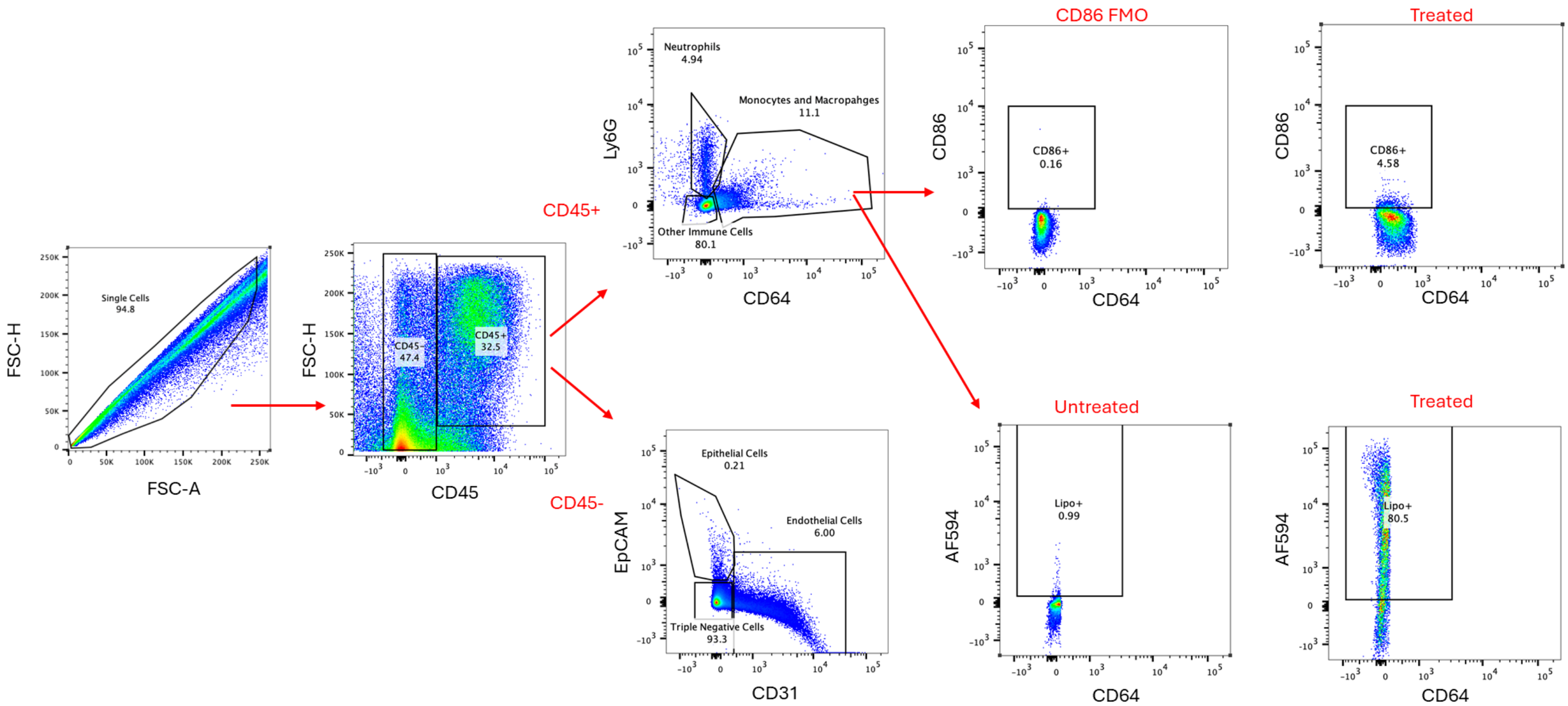

Supplementary Figure 16. Gating strategy for flow cytometry. To identify the cellular uptake of fluorescent 2-stage mixing LNPs in the live and spleen, we first gated for single cells, then split the single cell population into CD45+ immune cells and CD45- non-immune cells. In the immune cell population, we identify neutrophils (Ly6G+), monocytes and macrophages (CD64+), and “other immune cells” (CD45+/Ly6G-/CD64-). We further gated for CD86+ “activated immune cells” in each group. In the non-immune cell population, we identify endothelial cells (CD31+), epithelial cells (EpCAM+), and other “triple negative” cells (CD45-/CD31-/EpCAM-). To identify the LNP+ cells in each gate and the total single cell population, a fluorescence minus one (FMO) control was performed by surface staining for all cell types previously described in the organs of an untreated mouse. We set the upper threshold for a LNP+ cell based on the Alexa Fluor 594 signal of the FMO negative control (bottom right of figure).

Supplementary Methods 1: Chemical Synthesis of JSH-23 Derivatives

General Remarks

All vacuum/argon flushes and flame drying techniques were performed using a Schlenk line, along with septa and needles (no Schlenk flasks or multi-neck flasks were used, unless specifically stated). Reagents were used as obtained from commercial suppliers without further purification, unless otherwise noted. Anhydrous tetrahydrofuran and methylene chloride were dispensed from the solvent purification system, under argon atmosphere. Anhydrous dimethylformamide was purchased from Fisher. Microwave reaction vials were purchased from VWR. Celite 545 was purchased from EMD. ACS-grade hexanes, toluene, ethyl acetate, DCM, methanol, and diethyl ether were used for chromatography. Thin-layer chromatography (TLC) was performed on pre-coated silica gel 60 F254 glass-supported plates from EMD, and visualization was performed with a UV lamp followed by staining. Column chromatography was carried out on EM Science silica gel (60 Å pore size, 230-400 mesh). Deuterated solvents were purchased from Cambridge Isotope Laboratories. Potassium carbonate was flame-dried in a flask under vacuum, let to cool to room temperature, filled with argon, and as such added to bottles of deuterated chloroform to remove any acids formed due to the spontaneous decomposition of chloroform in the presence of light and oxygen. In case when water was observed in the ^{1}H NMR, 4 Å molecular sieves, previously activated by flame drying with a propane torch under vacuum, were added to the $CDCl_3$ bottle, and the bottle was left to stand for at least 24 h before use. For the preparation of NMR samples, about 0.70-0.75 mL of $CDCl_3/K_2CO_3$, measured with a glass Pasteur pipette attached to a 1 mL syringe or a 1 mL automatic pipette via a short piece of hose, was filtered through a small pad of cotton (placed in a second Pasteur pipette), and a pipette bulb was used to push out the residual chloroform from the cotton pad. Other deuterated solvents were used as received.

^{1}H and ^{13}C NMR spectra were recorded at room temperature on a 400, 500, or 600 MHz Bruker spectrometer and processed in MNova v15.0.1. Chemical shifts are given in parts per million (ppm) referenced to solvent residual proton resonance. NMR data are reported as: chemical shift, multiplicity (s = singlet, d = doublet, t = triplet, q = quartet, p = pentet/quintet, m = multiplet, and any combination of d, t, q, and p e.g., dt = doublet of triplets), coupling constants (*J*) given in Hz, and integration. In case when diastereomers are present, peaks corresponding to all isomers are listed. Compounds with complete spectral characterization already reported in the literature were only characterized by ^{1}H NMR for structure confirmation. Reactive intermediates were not spectrally characterized.

High resolution mass spectrometry was performed using the Bruker scimaX 2xR instrument at the Mass Spectrometry Facility at the Chemistry Department of the University of Pennsylvania.

CLogP: 10.261

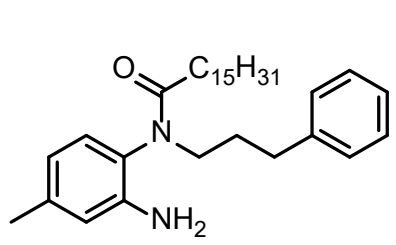

AM-1-049

CLogP: 19.841

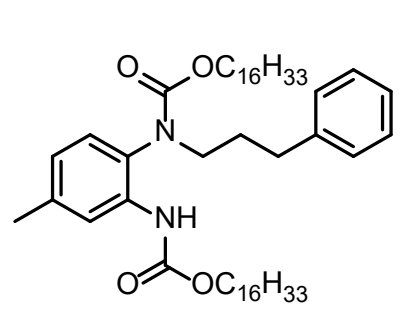

AM-1-055

CLogP: 12.02

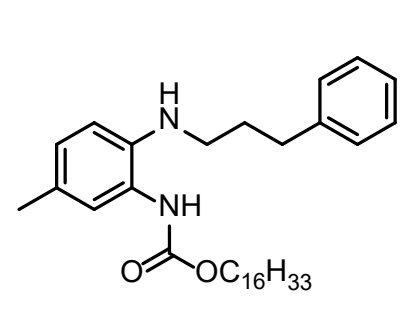

AM-1-061A

CLogP: 11.21

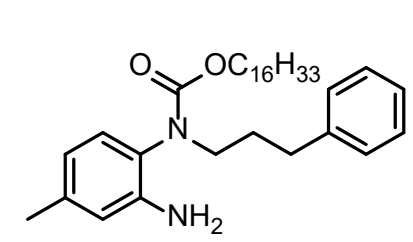

AM-1-061B

CLogP: 13.559

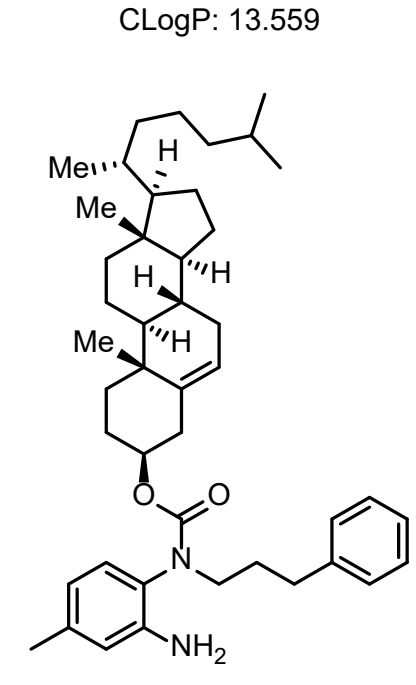

AM-1-078

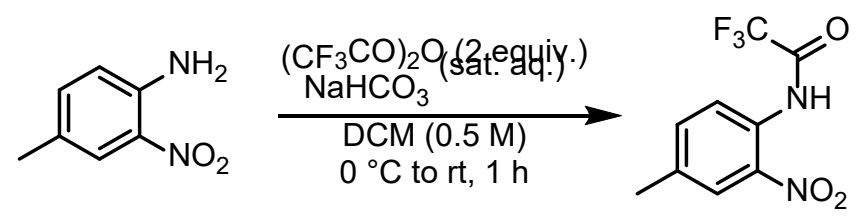

AM-1-037

To a round bottom flask, equipped with a stir bar, 4-methyl-2-nitroaniline (3.04 g, 20 mmol, 1 equiv.) and DCM (40 mL) were added. The mixture was stirred and cooled in an ice bath. Then, trifluoroacetic anhydride (5.6 mL, 2 equiv.) was added to the mixture dropwise. Next, saturated aqueous $NaHCO_3$ (20 mL) was added in one portion. The flask was closed with a cap and the mixture was stirred in the ice bath that was allowed to melt for 2 hours. Quench: The mixture was diluted with DCM and the layers were separated. Aqueous layer was back-extracted three times with DCM. Combined organic layers were dried over anhydrous $MgSO_4$, filtered, and the solvent was removed via a rotovap. The crude product AM-1-037 was directly used in the next step.

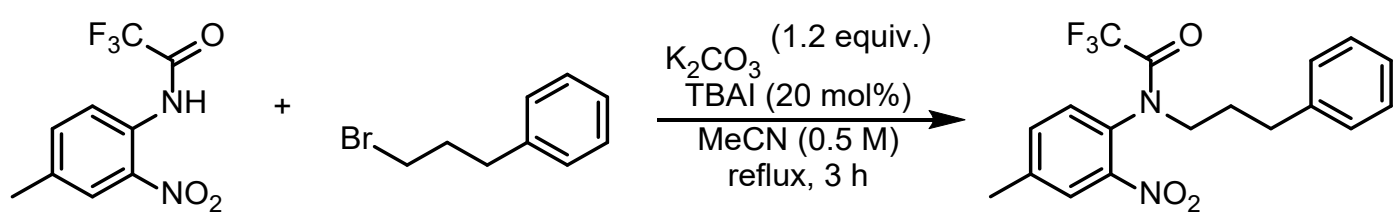

(1 equiv.) **AM-1-037** (1.3 equiv.) 85% over two steps **AM-1-040**

To a round-bottom flask, equipped with a stir bar, the crude nitro-trifluoroacetyl-aniline (20 mmol, 1 equiv.), anhydrous K_2CO_3 (3.32 g, 1.2 equiv.), TBAI (1.48 g, 20 mol%), and anhydrous acetonitrile (40 mL) were added. The mixture was stirred and then 1-bromo-3-phenylpropane (4 mL, 1.3 equiv.) was added dropwise. The mixture was stirred and refluxed for 3 h, and then left to stir at room temperature overnight. Quench: The mixture was diluted with Et_2O (~ 200 mL) and filtered over celite. The filtrate was concentrated via a rotovap. The crude material was redissolved in Et_2O and washed twice with water, then with saturated aqueous $NaHCO_3$. The organic layer was dried over anhydrous Na_2SO_4, and the solvent was removed via a rotovap. The crude product was purified via flash column chromatography on SiO_2 (~ 300 g, 10% EtOAc in hexanes). The pure product is an orange oil (6.25 g, 85%).

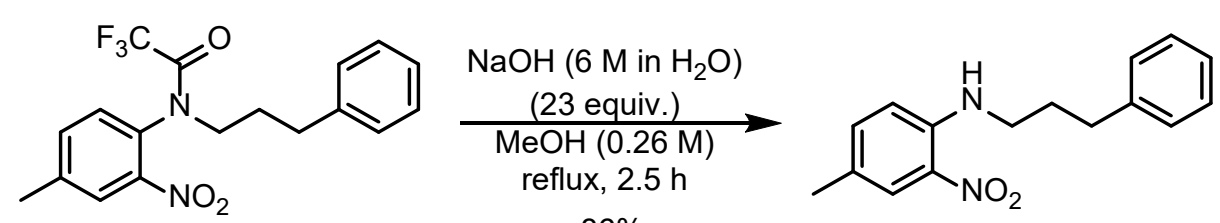

AM-1-040 86% **AM-1-045**

To a round-bottom flask, containing the trifluoroacetamide (6.25 g, 17.1 mmol, 1 equiv.) and a stir bar, methanol (65 mL) was added. The mixture was stirred and 6 M aqueous NaOH (65 mL, 23 equiv. of NaOH) was added in one portion. The mixture immediately changed color from orange to red. The reaction mixture was stirred and refluxed for 3.5 hours, after which it was left to stir at room temperature overnight. Quench: The mixture was diluted with a large volume of DCM (~ 250 mL) and then washed with water. The aqueous layer was back-extracted three times with DCM. The combined organic layers were dried over anhydrous Na_2SO_4, filtered, and the solvent was removed via a rotovap. The crude mixture was purified via flash column chromatography on SiO_2 (~ 120 g, 5% EtOAc in hexanes). The pure product is an orange-red liquid (3.966 g, 86%).

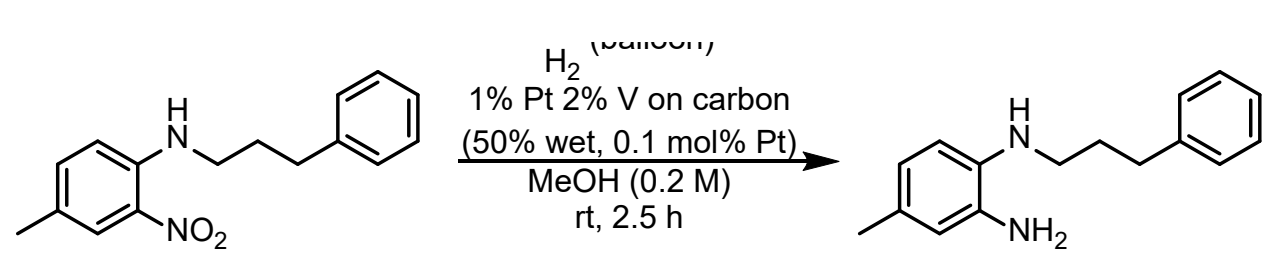

AM-1-045 90% **AM-1-048**

To a round-bottom flask, containing the nitroaniline derivative (3.966 g, 14.67 mmol, 1 equiv.) and a stir bar, methanol (70 mL) was added. The flask was closed with a septum and connected to a vacuum line via a needle. The mixture was vigorously stirred and briefly exposed to vacuum until solvent started boiling, then closed to the vacuum, and quickly punctured by another needle connected to a H_2 gas-filled double balloon. The H_2 balloon was then removed and the process was repeated two more times. The mixture was stirred under the atmosphere of hydrogen for 2.5 h, when TLC analysis (20% EtOAc in hexanes, R_f = 0.5 for nitroaniline, 0.2 for product aminoaniline) showed the reaction was complete. Quench: The mixture was filtered through celite, washing with methanol. A second celite filtration was attempted, however no material was retained on celite. The solvent was removed via a rotovap, and the obtained crude material was purified via flash column chromatography on SiO_2 (~ 120 g, 20% EtOAc in hexanes). The product crystallized out of the first few fractions containing the product. The product is a lightly plum-colored solid (3.158 g, 90%).

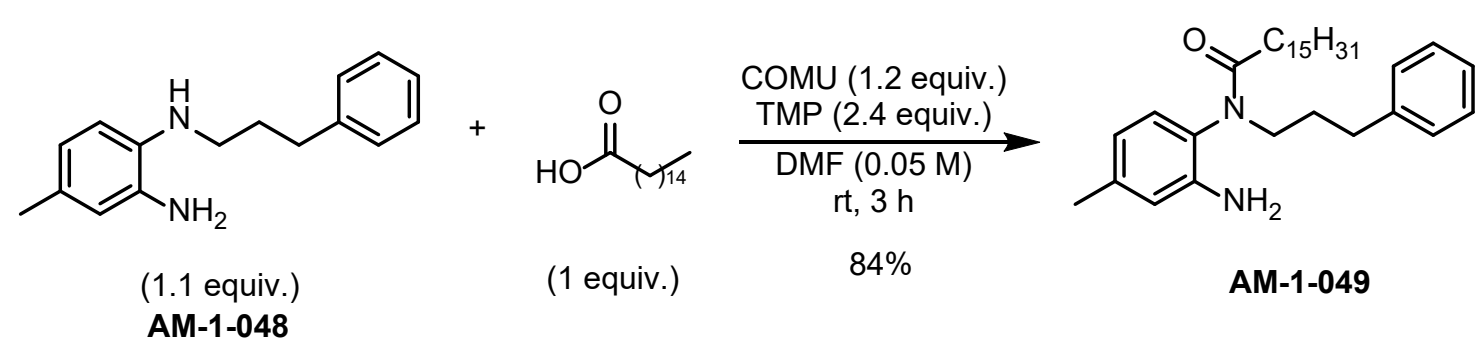

To a vial equipped with a stir bar, palmitic acid (26 mg, 0.1 mmol, 1 equiv.), JSH-23 (26 mg, 1.1 equiv.), and COMU (51 mg, 1.2 equiv.) were added. The mixture was dissolved in anhydrous DMF (2 mL) and stirred at room temperature. To the mixture, 2,4,6-collidine (32 µL, 2.4 equiv.) was added, and the reaction mixture was stirred at room temperature for 3 hours. Over this time, the appearance of the reaction mixture changed from homogeneous to a sludge. The progress of the reaction can be monitored by normal phase TLC (mini workup of an aliquot of the reaction mixture is diluted with some Et_2O and washed with some water), using 20% EtOAc in hexanes (product appears slightly above the reactant amine). Quench: The mixture was diluted with excess Et_2O and washed three times with water, then with saturated aqueous $NaHCO_3$, then with brine. The organic layer was dried over anhydrous Na_2SO_4, filtered, and the solvent was removed via a rotovap. The crude mixture was purified via flash column chromatography on SiO_2 (~ 4 g, 20% EtOAc in hexanes), to obtain a tan solid (40 mg, 84%).

^{1}H NMR (400 MHz, $CDCl_3$) δ 7.33 – 7.11 (m, 7H), 7.04 – 6.78 (m, 1H), 6.72 – 6.52 (m, 1H), 3.84 (s, 1H), 3.19 – 3.02 (m, 2H), 2.81 – 2.67 (m, 2H), 2.36 (t, J = 7.6 Hz, 2H), 2.24 (s, 3H), 2.13 – 1.88 (m, 2H), 1.80 – 1.50 (m, 2H), 1.27 (s, 28H), 0.89 (t, J = 6.7 Hz, 3H).

^{13}C NMR (101 MHz, $CDCl_3$) δ 172.23, 141.81, 139.30, 128.53, 128.52, 127.56, 126.03, 125.63, 125.11, 114.35, 44.51, 37.34, 33.60, 32.05, 31.13, 29.82, 29.78, 29.77, 29.66, 29.54, 29.49, 29.46, 26.08, 22.81, 20.57, 14.25.

HRMS (ESI) formula: $C_{32}H_{50}N_2O$ $[M+H]^+$ calc'd: 479.3996, found: 479.3994.

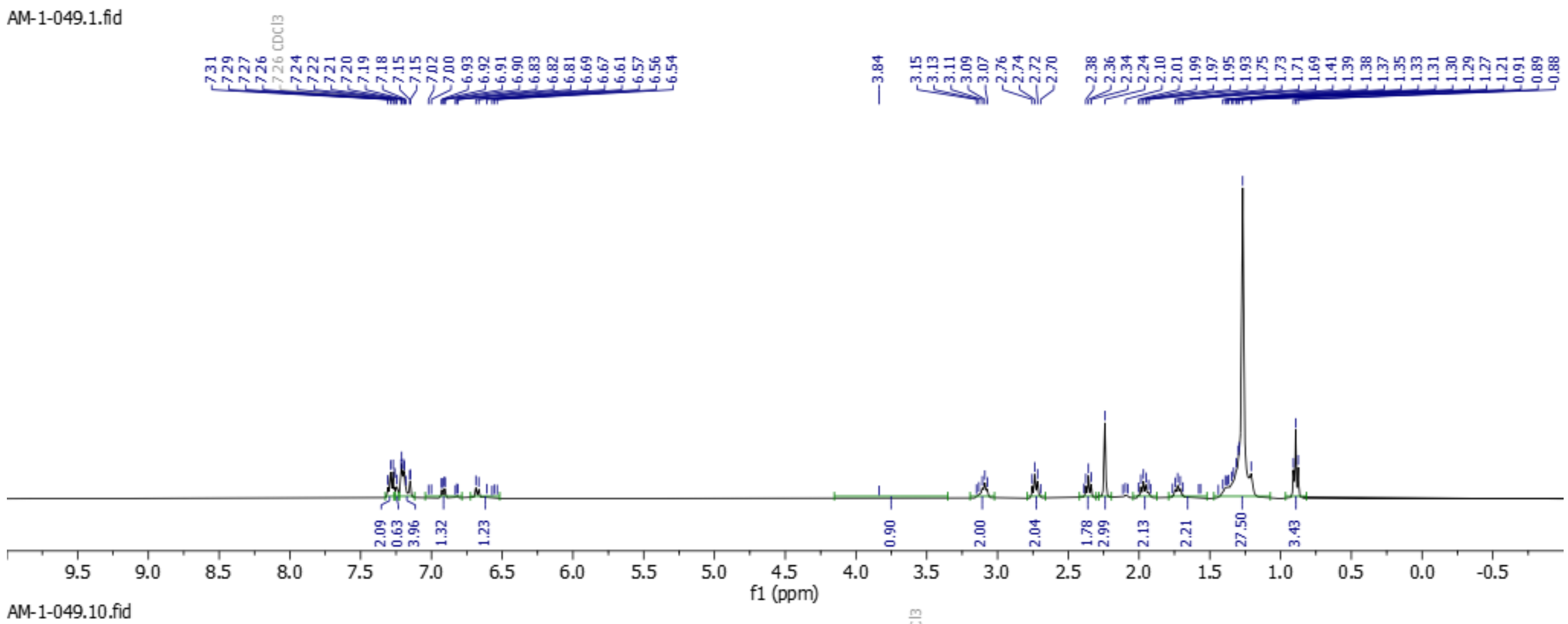

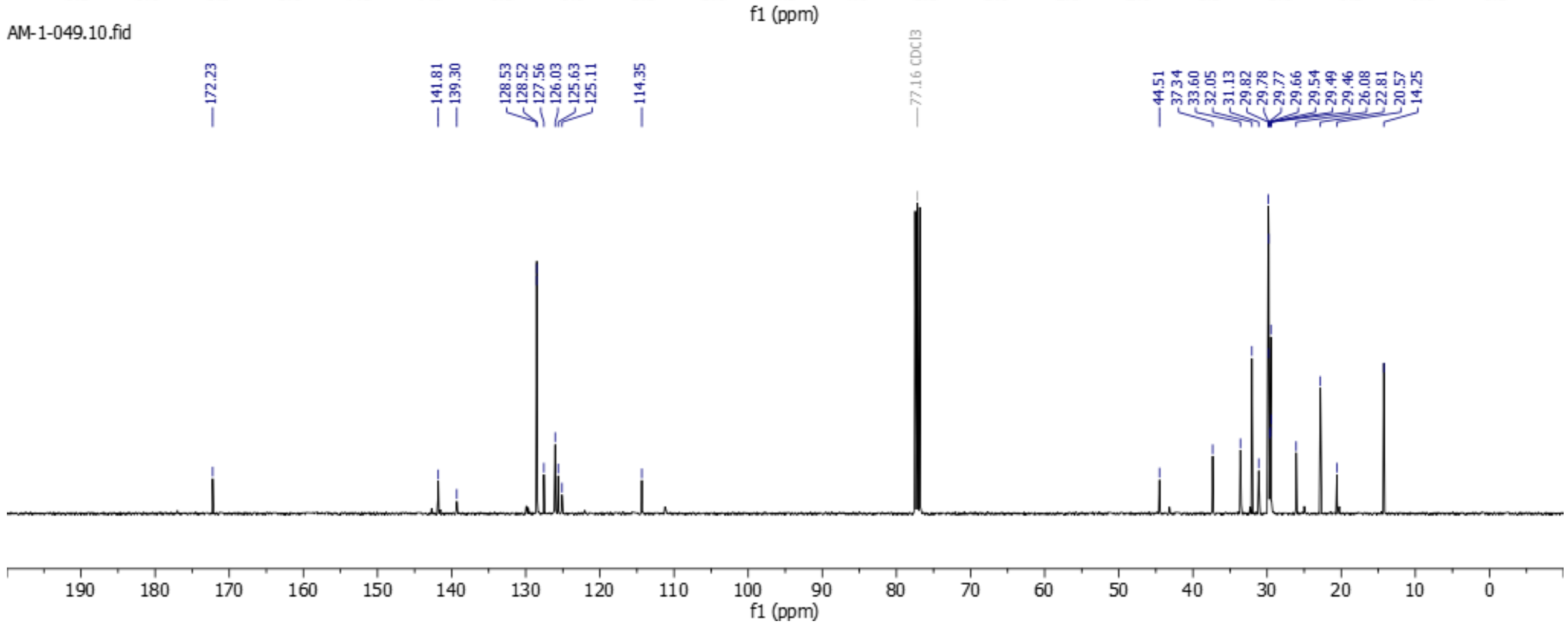

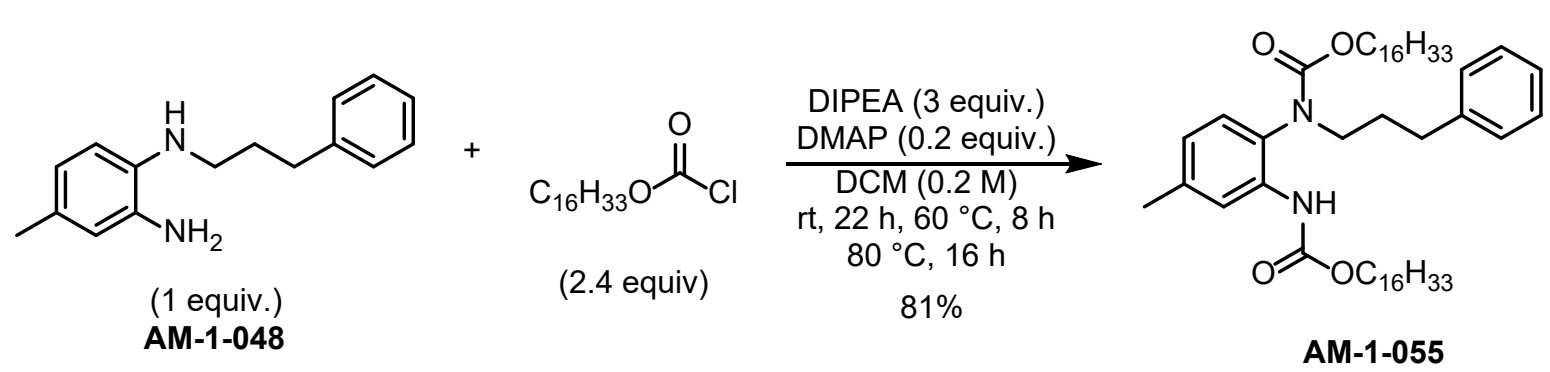

A microwave vial, equipped with a stir bar, was flame dried under vacuum / Ar. To the vial, 4-methyl-N1-(3-phenylpropyl)benzene-1,2-diamine (48 mg, 0.2 mmol, 1 equiv.), DMAP (5 mg, 0.2 equiv.), anhydrous DCM (1 mL), and DIPEA (0.10 mL, 3 equiv.) were added. To the stirring purple mixture, hexadecyl carbonochloridate (0.16 mL, 2.4 equiv.) was added, and an immediate color change was observed (purple to orange). The vial was crimped and the mixture was stirred at room temperature. After 22 hours, TLC analysis (20% EtOAc in hexanes) indicated partial conversion and formation of mono-carbamate regioisomers and potentially bis-carbamate. The vial was crimped again and heated to 60 °C with stirring. After 8 hours, TLC analysis of the mixture indicated that more of the products had formed, but the conversion was not complete. The vial was crimped again and the mixture was heated to 80 °C and stirred. After 16 hours, the mixture was diluted with DCM and washed once with saturated aqueous $NaHCO_3$. The organic layer was dried over anhydrous Na_2SO_4, filtered, and the solvent was removed via a rotovap. The crude mixture weighed 177 mg. The crude mixture was purified via flash column chromatography on SiO_2 (~ 10 g, 5% EtOAc in hexanes). The pure product is a colorless oil. Pure fraction = 83 mg (53%). Mixed fraction = 43 mg (28%). Total = 126 mg (81%).

^{1}H NMR (500 MHz, $CDCl_3$) δ 7.89 (br s, 1H), 7.25 (t, J = 7.5 Hz, 2H), 7.17 (t, J = 7.4 Hz, 1H), 7.13 (d, J = 7.4 Hz, 2H), 6.96 (d, J = 8.1 Hz, 1H), 6.87 (d, J = 8.0 Hz, 1H), 6.71 (br s, 1H), 4.16 – 4.04 (m, 4H), 3.69 – 3.50 (m, 2H), 2.59 (t, J = 7.6 Hz, 2H), 2.37 (s, 3H), 1.86 (p, J = 6.6 Hz, 2H), 1.65 (p, J = 6.8 Hz, 2H), 1.54 (br s, 2H), 1.42 – 1.11 (m, 52H), 0.89 (t, J = 6.8 Hz, 6H).

^{13}C NMR (126 MHz, $CDCl_3$) δ 156.17, 153.78, 141.34, 138.52, 134.74, 128.47, 128.37, 127.81, 126.04, 66.30, 65.68, 50.00, 33.22, 32.05, 29.83, 29.80, 29.75, 29.69, 29.65, 29.49, 29.43, 29.29, 29.23, 29.02, 28.97, 25.98, 25.86, 22.81, 21.58, 14.23.

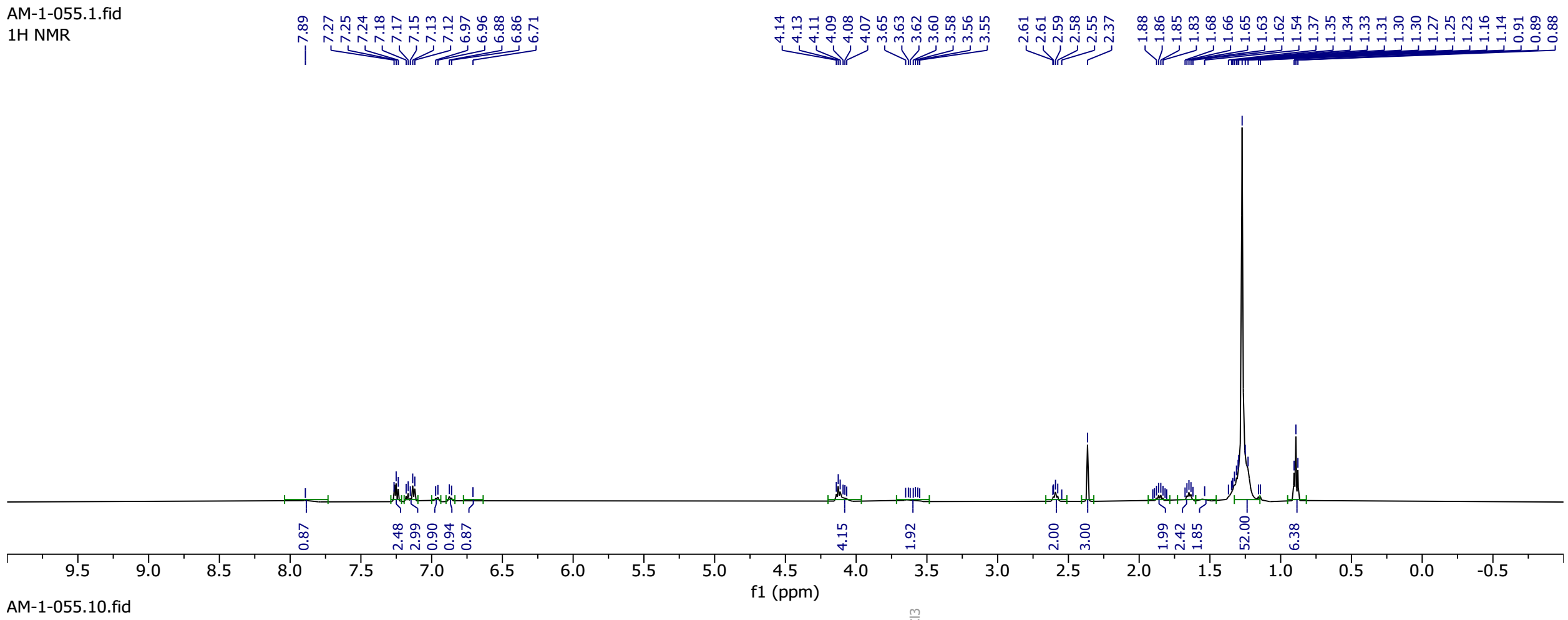

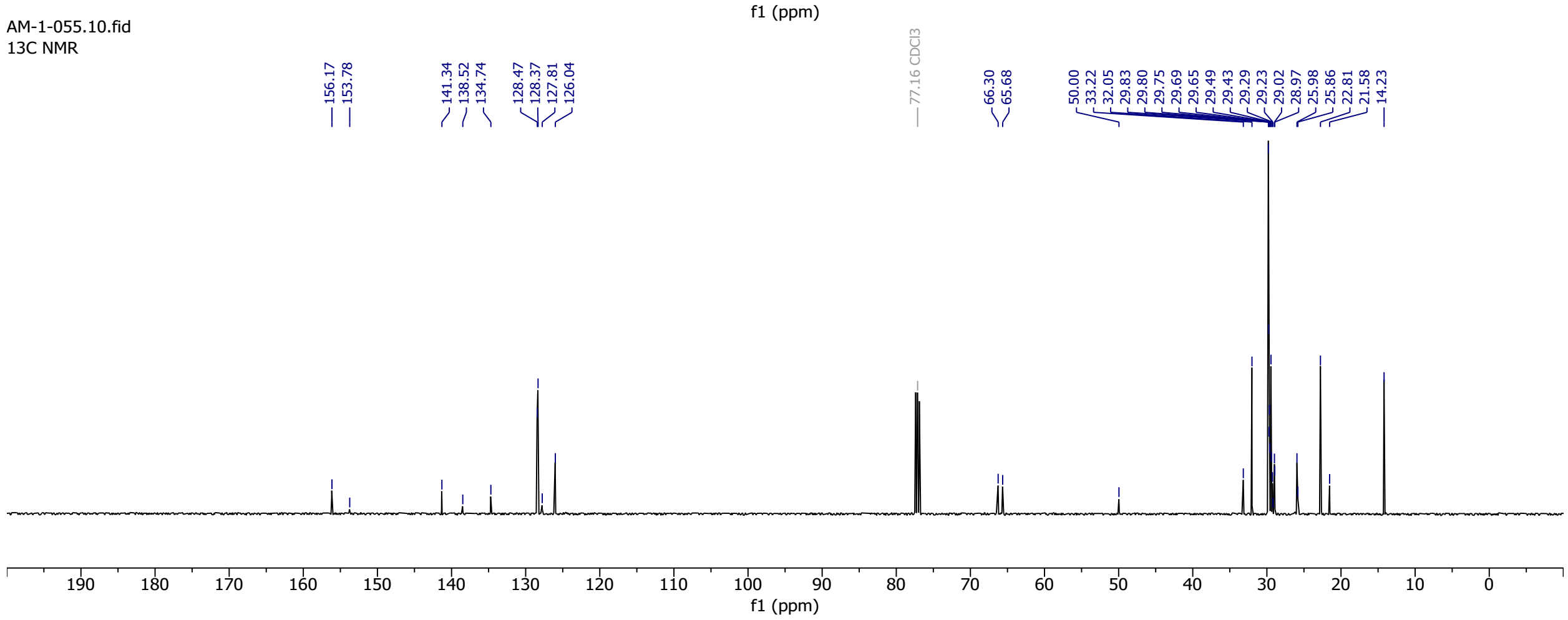

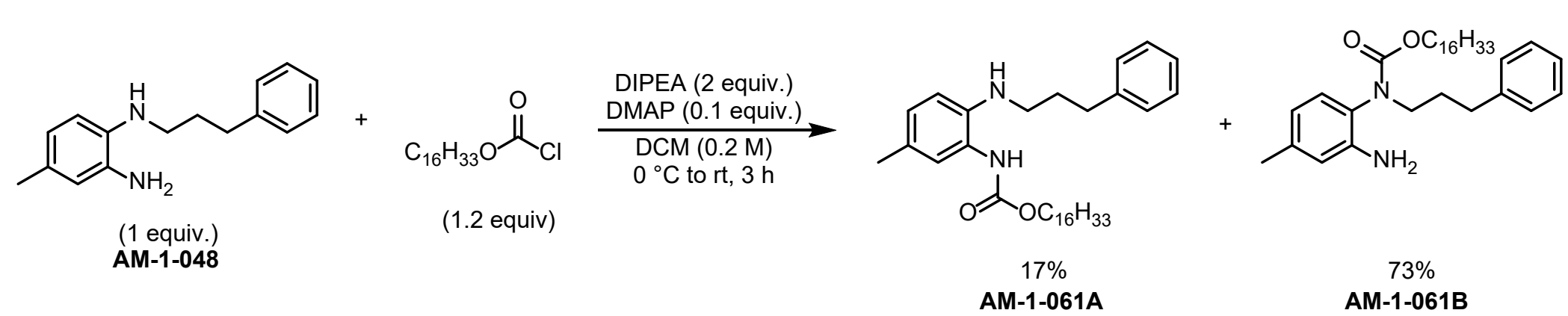

A vial, equipped with a stir bar, was flame dried under vacuum / Ar. To the vial, JSH-23 (96 mg, 0.4 mmol, 1 equiv.), DMAP (5 mg, 10 mol%), anhydrous DCM (2 mL), and DIPEA (0.14 mL, 2 equiv.) were added. The mixture was stirred under Ar and cooled to 0 °C with an ice/water bath. After 5 minutes, hexadecyl chloroformate (0.16 mL, 1.2 equiv.) was added dropwise, and the mixture was left to stir in a slowly melting ice/water bath. After three hours, the reaction mixture was diluted with Et_2O and washed three times with saturated aqueous $NaHCO_3$. The organic layer

was dried over anhydrous Na_2SO_4, filtered, and the solvent was removed via a rotovap. The crude mixture was purified via flash column chromatography on SiO_2 (~ 10 g, hexanes, then 5% EtOAc in hexanes, then 10%, then 15%). Two products were obtained, A (eluted with 5-10% EtOAc in hexanes; 35 mg, 17%, white solid), and B (eluted with 15% EtOAc in hexanes; 149 mg, 73%, pale red oil).

A:

^1H NMR (600 MHz, $CDCl_3$) δ 7.31 – 7.27 (m, 2H), 7.23 – 7.18 (m, 4H), 6.92 – 6.87 (m, 1H), 6.63 (d, J = 8.1 Hz, 1H), 6.28 (br s, 1H), 4.15 (t, J = 6.8 Hz, 2H), 3.11 (t, J = 7.0 Hz, 2H), 2.75 (t, J = 7.6 Hz, 2H), 2.25 (s, 3H), 2.01 – 1.93 (m, 2H), 1.67 (p, J = 6.9 Hz, 2H), 1.41 – 1.35 (m, 2H), 1.35 – 1.20 (m, 25H), 0.90 (t, J = 7.0 Hz, 3H).

^{13}C NMR (151 MHz, $CDCl_3$) δ 155.12, 141.82, 139.21, 128.55, 128.53, 126.96, 126.06, 124.92, 113.80, 65.76, 44.38, 33.61, 32.06, 31.17, 29.83, 29.82, 29.79, 29.74, 29.70, 29.50, 29.45, 29.12, 26.02, 22.82, 20.62, 14.26.

B:

^1H NMR (600 MHz, $CDCl_3$) δ 7.30 – 7.24 (m, 2H), 7.19 – 7.16 (m, 3H), 6.88 (br s, 1H), 6.62 – 6.54 (m, 2H), 4.12 – 4.09 (m, 2H), 3.86 – 3.42 (m, 4H), 2.67 – 2.59 (m, 2H), 2.29 (s, 3H), 2.00 – 1.85 (m, 2H), 1.76 – 1.48 (m, 2H), 1.43 – 1.14 (m, 26H), 0.92 (t, J = 7.0 Hz, 3H).

^{13}C NMR (151 MHz, $CDCl_3$) δ 156.36, 142.85, 141.73, 138.29, 128.88, 128.57, 128.55, 128.39, 128.30, 128.20, 128.18, 126.04, 125.89, 125.67, 124.95, 119.48, 116.81, 65.88, 49.07, 33.28, 32.01, 30.12, 29.79, 29.78, 29.76, 29.75, 29.63, 29.45, 29.27, 29.04, 25.80, 22.78, 21.27, 14.21.

AM-1-061A.1.fid

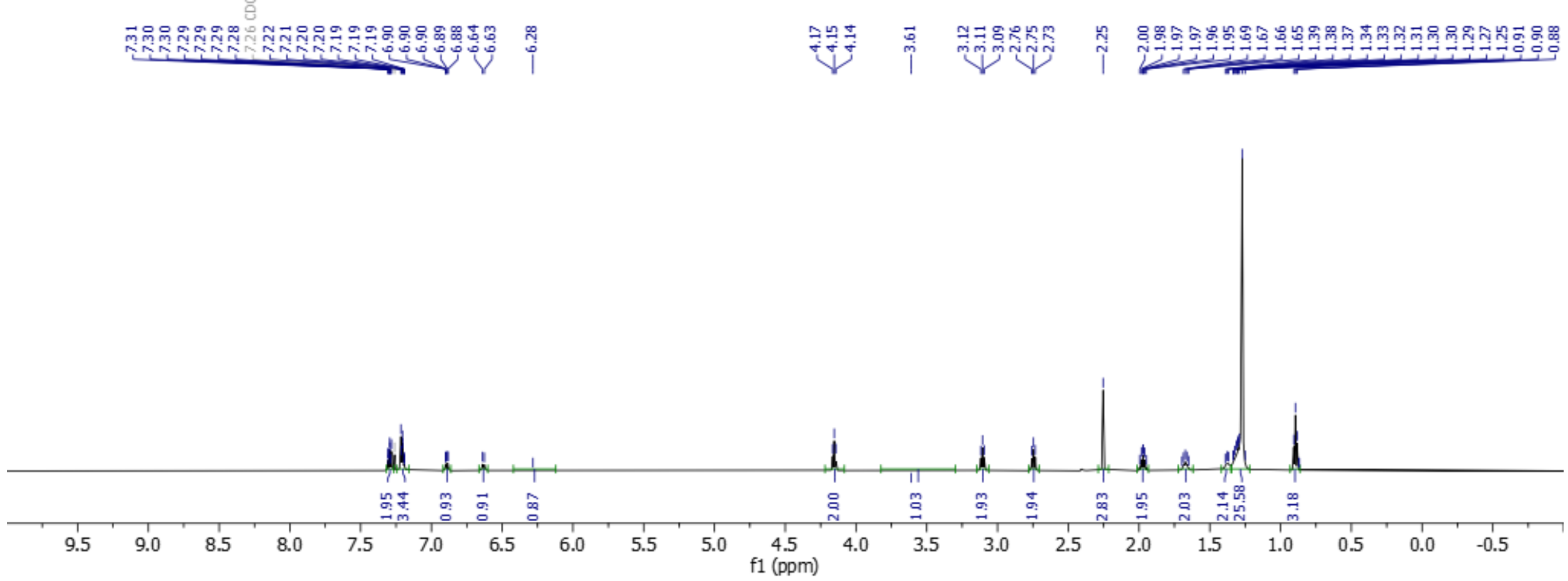

AM-1-061A.10.fid

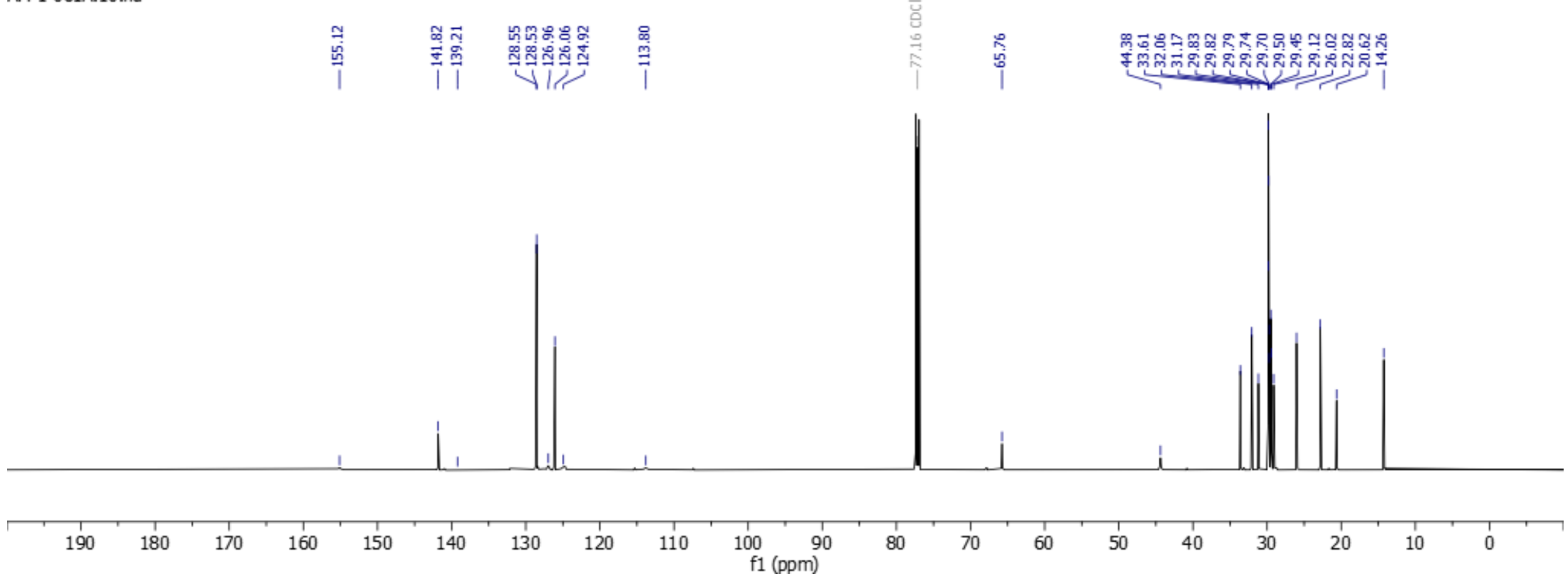

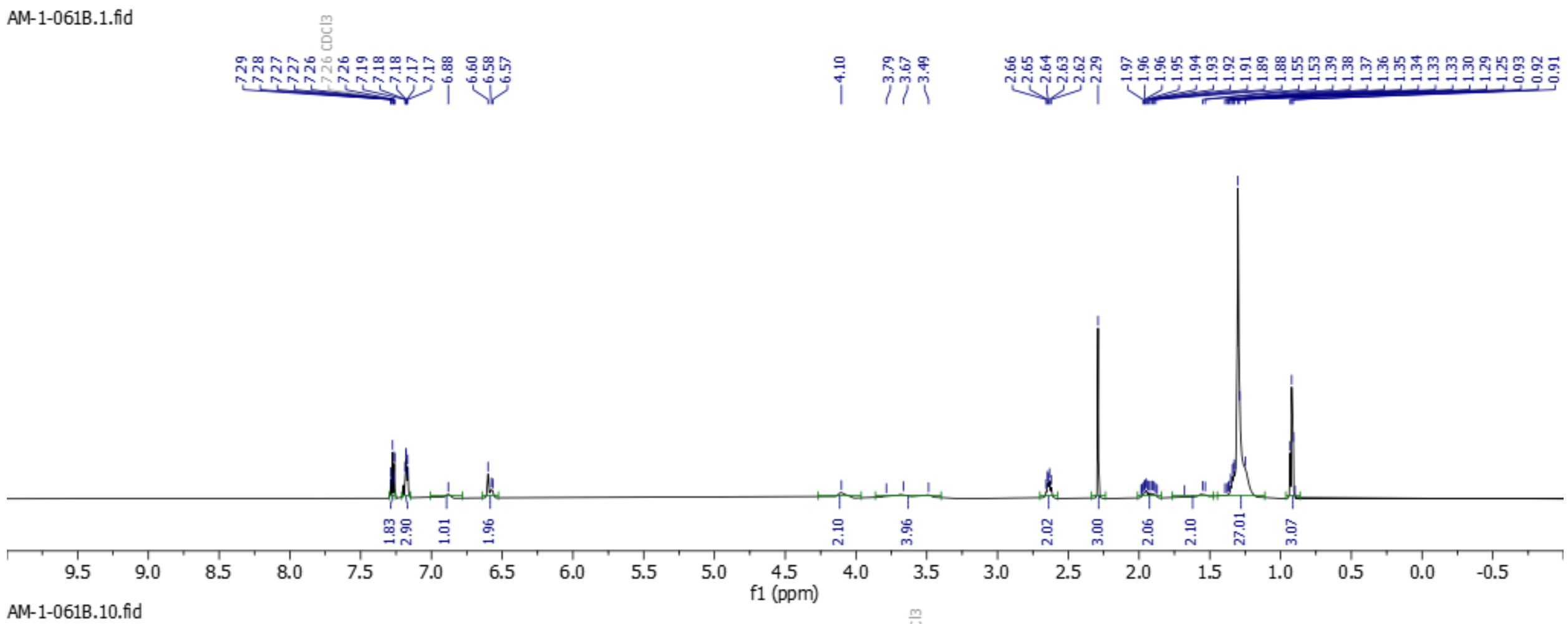

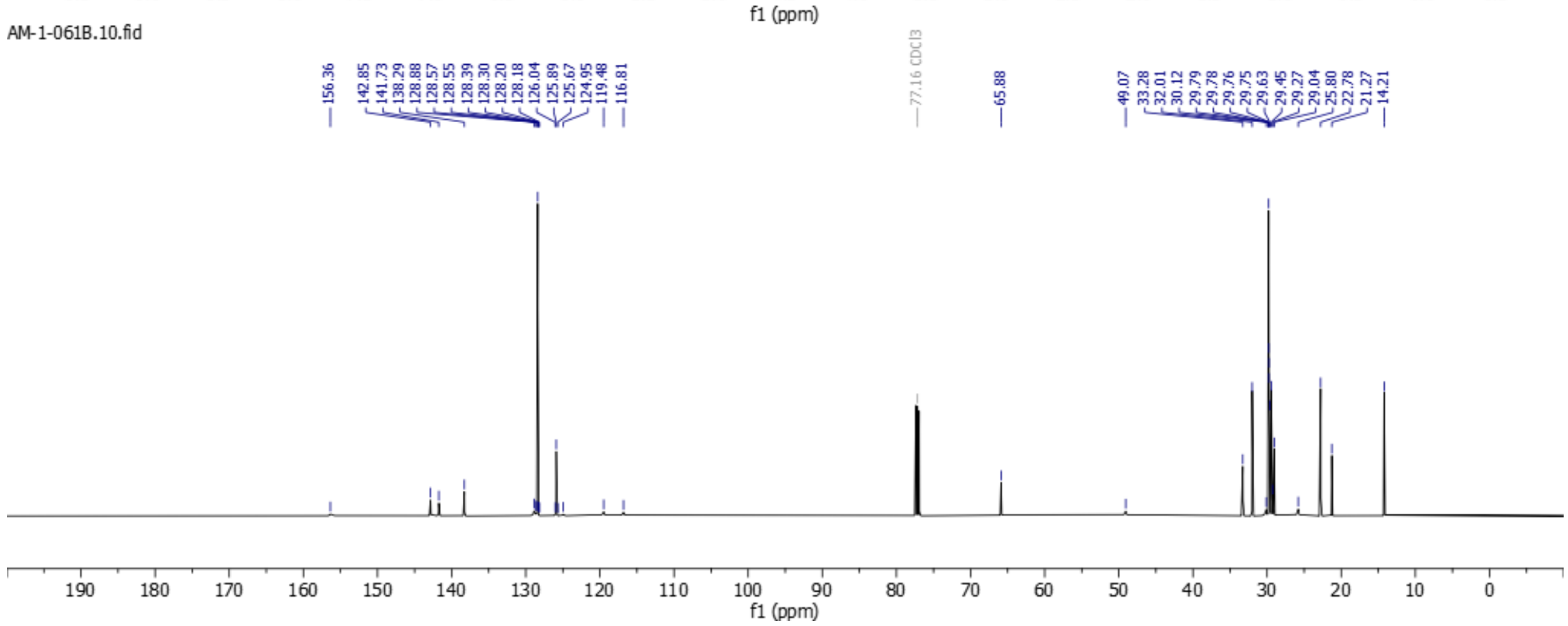

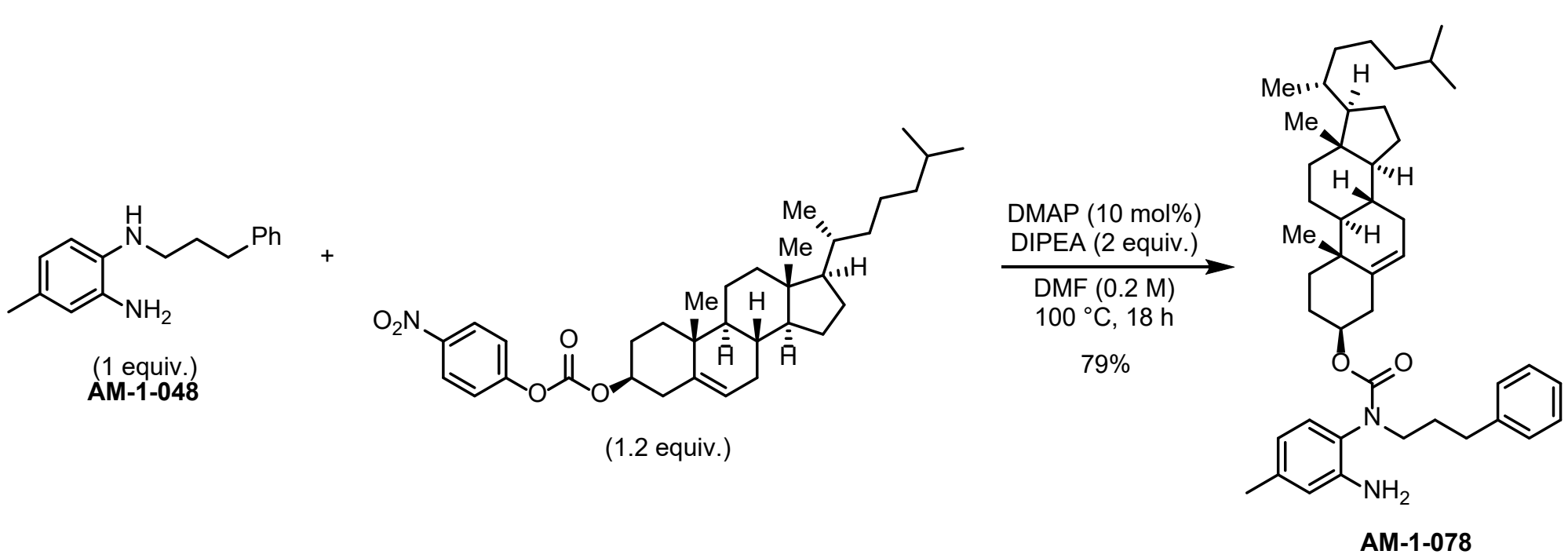

To a vial, equipped with a stir bar, JSH-23 (48 mg, 0.2 mmol, 1 equiv.), cholesterol PNP carbonate (132 mg, 1.2 equiv.), DMAP (3 mg, 10 mol%), anhydrous DMF (1 mL) and DIPEA (69 µL, 2 equiv.) were added. The vial was closed, and the mixture was stirred at 100 °C (oil bath). After 18 hours, the mixture was diluted with Et_2O and washed five times with saturated aqueous $NaHCO_3$, then with brine. The organic layer was dried over anhydrous Na_2SO_4, filtered, and the solvent was removed via a rotovap. The crude mixture was purified via flash column chromatography on SiO_2 (20 g, 5% EtOAc in hexanes). The pure product is a pale yellow oil / foam (103 mg, 79%).

^{1}H NMR (600 MHz, $CDCl_3$) δ 7.32 – 7.28 (m, 2H), 7.24 – 7.20 (m, 3H), 6.89 (dd, J = 8.2, 2.1 Hz, 1H), 6.64 (d, J = 8.1 Hz, 1H), 6.34 (br s, 1H), 5.47 – 5.36 (m, 1H), 4.67 – 4.58 (m, 1H), 3.57 (br s,

1H), 3.11 (t, J = 7.0 Hz, 2H), 2.76 (t, J = 7.6 Hz, 2H), 2.50 – 2.42 (m, 1H), 2.42 – 2.33 (m, 1H), 2.26 (s, 3H), 2.08 – 1.82 (m, 7H), 1.69 – 1.00 (m, 25H), 0.95 (d, J = 6.5 Hz, 3H), 0.91 (d, J = 2.7 Hz, 3H), 0.90 (d, J = 2.7 Hz, 3H), 0.71 (s, 3H).

^{13}C NMR (151 MHz, $CDCl_3$) δ 154.38, 141.82, 139.76, 139.06, 128.52, 128.51, 128.16, 126.72, 126.04, 125.06, 124.68, 122.79, 113.79, 75.18, 56.79, 56.25, 50.10, 44.34, 42.42, 39.85, 39.64, 38.58, 37.09, 36.68, 36.31, 35.92, 33.59, 32.02, 31.98, 31.19, 28.35, 28.21, 28.12, 24.40, 23.96, 22.95, 22.69, 21.16, 20.64, 19.45, 18.84, 11.98.

HRMS (ESI) formula: $C_{44}H_{64}N_2O_2$ $[M+H]^+$ calc'd: 653.5041, found: 653.5041.

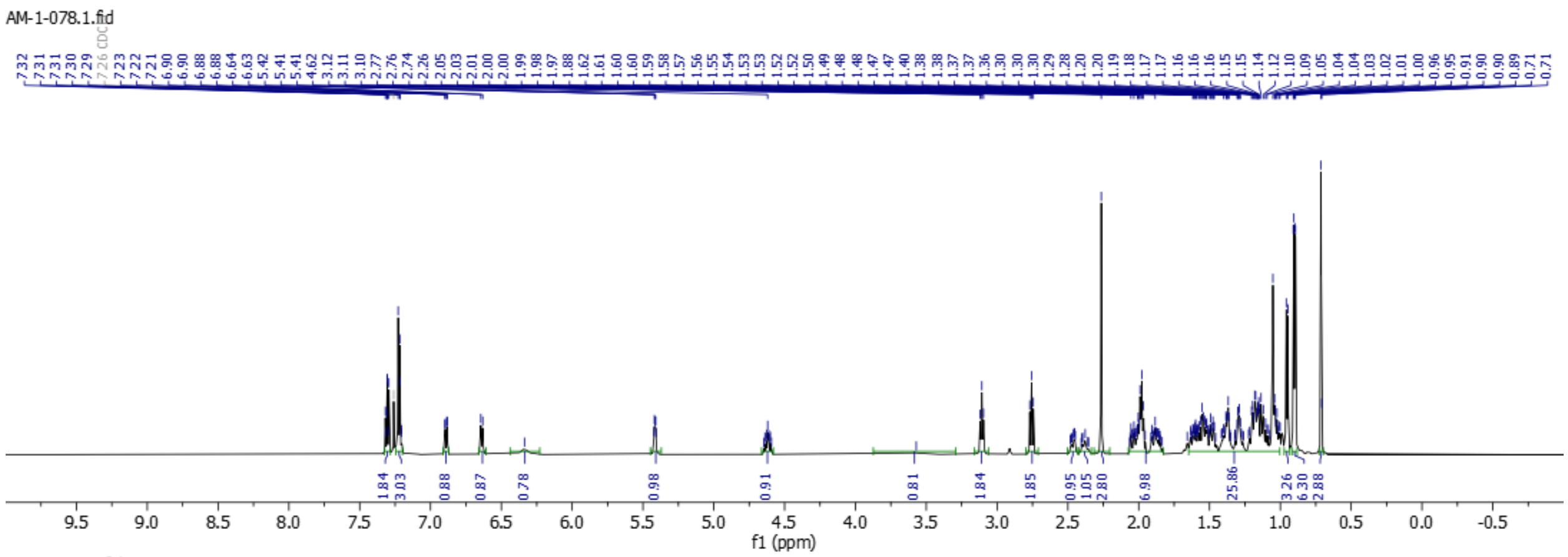

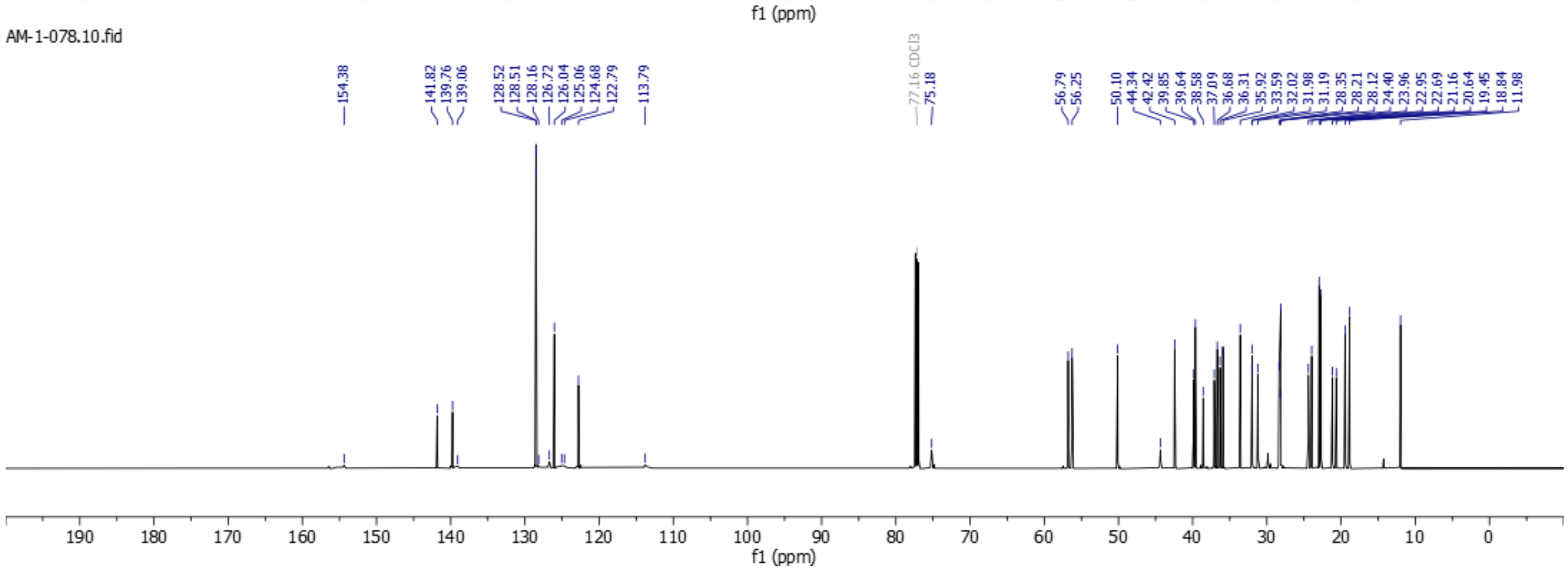

Supplement: Supplementary file 1 [file ADVS-13-e17931-s001.pdf]
